# A Combination of Geographically Weighted Regression, Particle Swarm Optimization and Support Vector Machine for Landslide Susceptibility Mapping: A Case Study at Wanzhou in the Three Gorges Area, China

**DOI:** 10.3390/ijerph13050487

**Published:** 2016-05-11

**Authors:** Xianyu Yu, Yi Wang, Ruiqing Niu, Youjian Hu

**Affiliations:** 1 Faculty of Information Engineering, China University of Geosciences, Wuhan 430074, China; yuxianyu1987@126.com (X.Y.); hyj_06@163.com (Y.H.); 2 Institute of Geophysics and Geomatics, China University of Geosciences, Wuhan 430074, China; rqniu@163.com

**Keywords:** landslide susceptibility mapping, geographically weighted regression, support vector machine, particle swarm optimization, Three Gorges Reservoir

## Abstract

In this study, a novel coupling model for landslide susceptibility mapping is presented. In practice, environmental factors may have different impacts at a local scale in study areas. To provide better predictions, a geographically weighted regression (GWR) technique is firstly used in our method to segment study areas into a series of prediction regions with appropriate sizes. Meanwhile, a support vector machine (SVM) classifier is exploited in each prediction region for landslide susceptibility mapping. To further improve the prediction performance, the particle swarm optimization (PSO) algorithm is used in the prediction regions to obtain optimal parameters for the SVM classifier. To evaluate the prediction performance of our model, several SVM-based prediction models are utilized for comparison on a study area of the Wanzhou district in the Three Gorges Reservoir. Experimental results, based on three objective quantitative measures and visual qualitative evaluation, indicate that our model can achieve better prediction accuracies and is more effective for landslide susceptibility mapping. For instance, our model can achieve an overall prediction accuracy of 91.10%, which is 7.8%–19.1% higher than the traditional SVM-based models. In addition, the obtained landslide susceptibility map by our model can demonstrate an intensive correlation between the classified very high-susceptibility zone and the previously investigated landslides.

## 1. Introduction

It is known that the area in the Three Gorges Reservoir along the Yangtze River is characterized by many active and reactivated landslides caused by the periodic water level fluctuation of the reservoir [1], which poses a serious threat to the security of life and property. Up to 2009, more than 3800 landslides have been recorded in this region [2]. Thus, it is crucial to predict slope failures in the Three Gorges area.

Landslide susceptibility evaluation is a complex task [3]. Compared to the traditional geological survey methods, such as landslide field reconnaissance, landslide spatial prediction is more convenient and efficient, due to the integration of geographical information systems (GIS) technology and statistical analysis principles. The spatial prediction of landslide susceptibility mapping is considered as one of the most important steps for landslide hazard mitigation and management [4], which has encouraged research towards knowledge-driven and data-driven models [5]. Knowledge-driven models, such as analytic hierarchy process (AHP) and fuzzy mathematics [5,6], are based on the analysis of landslide formation mechanism(s), and expert experience and knowledge are used to choose the most important environmental factors of landslides and quantitative weight values. On the other hand, data-driven models include logistic regression (LR) [7,8,9], artificial neural network (ANN) [10,11,12,13], SVM [14,15,16,17] and geographically weighted regression (GWR) [18,19], *etc.* These models are based on overlay analysis to calculate quantitative relationship between various environmental factors and the known distributions of landslides. Therefore, they are always used to determine weights of predictors, *i.e.*, values/indices of landslide susceptibility.

Since support vector machine (SVM) can demonstrate satisfactory classification accuracies when a limited number of training samples is available, and it has been widely utilized to perform landslide susceptibility mapping [14,15,16,17,20,21]. However, the proper selection of a kernel function and its corresponding parameters is still an open problem, which can greatly influence the final prediction accuracy. To obtain the optimal parameters for SVM, some researchers worked on combining the particle swarm optimization (PSO) algorithm with the classical SVM model [22,23,24]. PSO is a population-based stochastic optimization technique developed by Eberhart and Kennedy [25], inspired by social behavior of bird flocking or fish schooling. This technique has many similarities with evolutionary computation techniques such as Genetic Algorithms (GA) [26]. For instance, the system is initialized with a population of random solutions and searches for optima by updating generations. However, unlike GA, PSO has no evolution operators such as crossover and mutation. In PSO, the potential solutions, called particles, fly through the problem space by following the current optimum particles. Compared to GA, the advantages of PSO are that it is easy to implement and there are few parameters to adjust [27]. To better perform landslide prediction, this technique can estimate optimum parameters for the SVM prediction model. For instance, Huang and Dun [22] proposed a PSO–SVM model to improve classification accuracies with an appropriate feature subset. One year later, Zhao and Yin [23] integrated the SVM, PSO and numerical analysis techniques to intelligent displacement back analysis in geomechanical parameter identification. More recently, Ren *et al.* [24] presented a landslide prediction method for the Shuping landslide by using a PSO-SVM model and wavelet analysis. However, the drawbacks of these techniques are threefold: first, the PSO algorithm always falls into a local optimum, especially in a very large area. Second, spatial autocorrelation in study areas is not taken into account. Finally, these methods applied a global model in a certain area and considered that the impacts of environmental factors are equal for the entire region, so they cannot describe the local characteristics of spatial landslide occurrences.

This paper presents an effective PSO-SVM model based on GWR for landslide susceptibility mapping. It should be noted that in practice different degrees of impact may occur at a local scale for study areas [18]. Moreover, the impacts of environmental factors always vary with spatial locations. It is well-known that most variables in real-world applications tend to be moderately spatially autocorrelated because of the way phenomena are geographically organized [28,29]. Therefore, spatial autocorrelation is always used to measure the degree to which a set of spatial features and their associated data values tend to be clustered together in space or dispersed [30,31]. Recently, many contributions have been devoted to using GWR to account for spatial autocorrelation and these have validated that GWR can be an effective estimator of spatial autocorrelation [32,33,34,35]. Inspired by previous works, we utilize the GWR technique to segment the study area into several prediction regions with a proper size. To this end, each computing unit in the study area is assigned a GWR coefficient by exploiting an appropriate kernel type and selection criteria. Meanwhile, each environmental factor is divided into several classes by the natural breaks method. By superposing these classification maps, different degrees of impacts at a local scale for these environmental factors are taken into account as well. As a consequence, the GWR coefficients in each prediction region are similar, while they make a great difference in different regions, *i.e.*, spatial autocorrelations of environmental factors between them are greatly suppressed. Secondly, the PSO-SVM model is used in each prediction region for landslide susceptibility mapping. The PSO algorithm is utilized for the SVM model to search for optimal parameters in each prediction region. In this way, the problem of local optimum can be effectively overcome. In addition, the SVM model can be locally applied to each prediction region for accurate landslide susceptibility maps.

The remainder of this paper is organized as follows: Section 2 reviews the related techniques on GWR, PSO and SVM. Section 3 presents the proposed GWR-PSO-SVM model. Section 4 describes the study area and data used in this work. Section 5 reports experiments including comparative results between the traditional SVM-based prediction models and ours. Section 6 presents some discussions of our model and the last section states our concluding remarks.

## 2. Related Techniques

### 2.1. Geographically Weighted Regression

Geographically Weighted Regression (GWR) is a fairly recent contribution to modelling spatially heterogeneous processes [28,29,36,37] that has attracted much attention for its elegant performance when exploring local variations in a study area [18,38,39]. GWR is implemented by obtaining regression equations for each spatial zone separately [40] and its basic model can be written as:

(1) $$y_{i}=\beta_{0}\left(u_{i},v_{i}\right)+\sum_{k=1}^{Q}\beta_{k}\left(u_{i},v_{i}\right)x_{ik}+\varepsilon_{i}$$

where $\left(u_{i},v_{i}\right)$ denotes the coordinates of the *i*th sample in space (e.g., latitude and longitude), *i* = 1,2,⋯,*L*, *L* and *Q* are the number of samples and regression coefficients, respectively. *y_i_* is the dependent variable at location *i*, *x_ik_* is the value of the *k*th explanatory variable at location *i*, $\beta_{k}(u_{i},v_{i})$ is the local regression coefficients for the *k*th explanatory variable at location *i*, and $\beta_{0}(u_{i},v_{i})$ is the intercept parameter at location *i*. Then, the least square estimate of *β_i_* can be defined as follows:

(2) $${\hat{\beta }}_{i}={\left(X^{T}W_{i}X\right)}^{-1}X^{T}W_{i}Y$$

and its variance is:

(3) $$\text{var}(\hat{\beta })={\left(X^{T}W_{i}^{-1}X\right)}^{-1}$$

where *W_i_* is n×n diagonal matrix, whose diagonal elements are the geographical weights:

(4) $$W_{i}=\left[\begin{array}{cccc} W_{i1} & 0 & \cdots & 0\\ 0 & W_{i2} & \cdots & 0\\ \vdots & \vdots & \ddots & \vdots \\ 0 & 0 & \cdots & W_{in} \end{array}\right]$$

the choice of *W_i_* depends on the selected kernel function, which can be fixed (*i.e.*, fixed bandwidth) or adaptive kernels (*i.e.*, varying bandwidths) in [41].

In practical, it is found that GWR is not sensitive to the choice of Gaussian function and bi-square function, but rather the bandwidth of the specific weight function. Based on the maximum likelihood principle, Akaike [42] proposed a general model selection criterion, called the Akaike Information Criterion (AIC), which is shown as follows:

(5) $$AIC=-2\text{ln}\;L\left({\hat{\theta }}_{L},x\right)+2q$$

where $L\left({\hat{\theta }}_{L},x\right)$ is the maximized likelihood of the parameter vector *θ*, *x* is a random sample, ${\hat{\theta }}_{L}$ is the maximum likelihood estimate of *θ*, *q* is the number of the unknown parameters. The larger the likelihood function, the better the estimator. In this work, a minimum AIC model is selected as the “optimal” model.

### 2.2. Support Vector Machine

**Figure 1:**
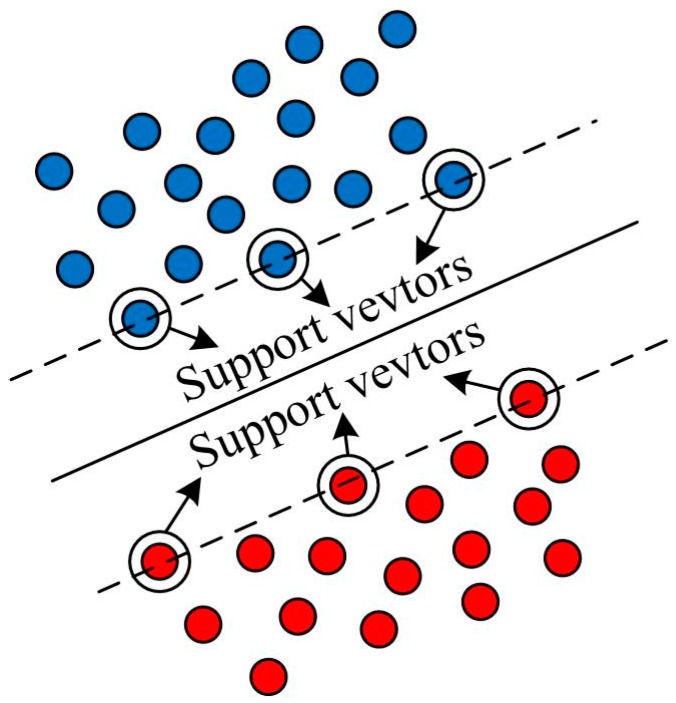
Illustration of the SVM principle.

The support vector machine (SVM) incorporates mainly two learning techniques [43], *i.e.*, Vapnik–Chervonenkis (VC) dimensional and statistical learning theories. One of the most important applications of SVM is classification. Because of its satisfactory performance and capabilities of fault-tolerance, SVM has recently attracted increasing attention and is widely used in machine learning, data mining and knowledge discovery [44,45], as well as landslide susceptibility assessment [14,15,16,17]. The SVM method is briefly introduced as follows [46,47]: assuming that a set of linear separable training vectors *x_i_*(*i* = 1,2,⋯,*R*, *R* is the total number of vectors) consists of two classes *y_i_* = ±1, which denote as landslide occurrence or not. The aim of SVM is to find an *n*-dimensional hyperplane to split two classes by the maximum gap, as shown in Figure 1. The *n*-dimensional hyperplane can be minimized as:

(6) $$\left\{ \begin{array}{l} \text{min}\frac{1}{2}{\left\|w\right\|}^{2},\\ s.t.,\;y_{i}\left(w\cdot x_{i}+b\right)\geq 1 \end{array}\right.$$

where ‖*w*‖ is the two-norm of *w*, *b* is used to increase the interval to ensure that the hyperplane does not cross the origin, *x_i_* is the point of the hyperplane, and *w* is a vector perpendicular to the hyperplane. By embedding a non-negative Lagrange multiplier (*λ_i_*), the cost function can be obtained as follows:

(7) $$L\left(w,b,\lambda \right)=\frac{1}{2}{\left\|w\right\|}^{2}-\sum_{i=1}^{n}\lambda_{i}\left(y_{i}\left(w\cdot x_{i}+b\right)-1\right)$$

The solution can be obtained by dual minimizing Equation (7) with respect to *w* and *b*. In the non-separable case, one can complete the constraints by introducing a non-negative *ξ_i_*, then Equation (7) can be produced as follows:

(8) $$\left\{ \begin{array}{l} \text{min}\frac{1}{2}{\left\|w\right\|}^{2}+C\sum_{i=1}^{n}\xi_{i},\\ s.t.,\;y_{i}\left(\left(w\cdot x_{i}\right)+b\right)\geq 1-\xi_{i} \end{array}\right.$$

where *ξ_i_*(*ξ_i_* ≥ 0) is the slack variable, *C* is a penalty variable of the error term, which denotes the distance from a wrong point to its correct position.

In addition, the Gaussian Radial Basis Function (RBF) is used as a kernel function introduced by Vapnik [43] to account for the nonlinear decision boundary:

(9) $$K(x_{i},x_{j})=\text{exp}(-\gamma {\left\|x_{i}-x_{j}\right\|}^{2})$$

where *γ* is a positive variable to measure the width of the Gaussian kernel in RBF. This function is robust and can account for the nonlinear decision boundary.

### 2.3. Particle Swarm Optimization

**Figure 2:**
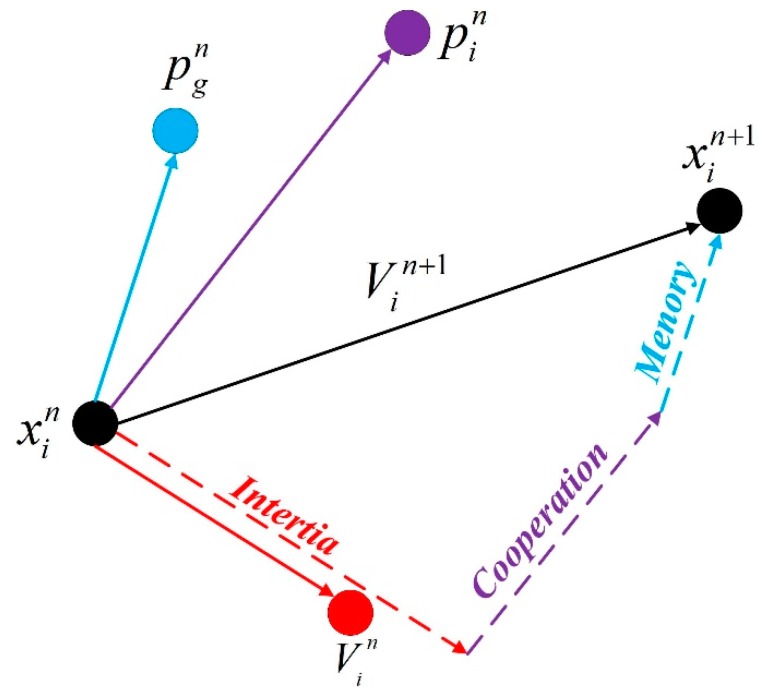
The process of the PSO algorithm.

The PSO algorithm is an evolutionary computation technique [25], which is derived from the complex adaptive system (CAS). The algorithm was originally inspired by the regularity of the activity of birds, and then a simplified model was established based on swarm intelligence. In PSO, the solution of each optimization problem is a bird in the search space, called a “particle”. PSO is initialized to a group of random particles and used to search the optimal solution by iterative evolution. In each iteration, the particles update themselves by tracking extremes of velocity and position. The above-mentioned behavior of the *i*th particles can be mathematically expressed as follows [48]:

(10) $$\{\begin{array}{l} V_{i}^{n+1}=t\cdot V_{i}^{n}+c_{1}\cdot r_{1}\cdot (p_{i}^{n}-x_{i}^{n})+c_{2}\cdot r_{2}\cdot (p_{g}^{n}-x_{i}^{n})\\ x_{i}^{n+1}=x_{i}^{n}+V_{i}^{n} \end{array}$$

where *i* = 1, 2, ⋯, *K*, *K* is the total number of particles, *n* is the current number of iteration. *t* is the inertia weight, $p_{i}^{n}$ and $p_{g}^{n}$ are the individual optimal position of the *i*th particle and the optimal position of all particles at the iteration of *n*, respectively. $V_{i}^{n}$ and $x_{i}^{n}$ are the velocity and the current position of the *i*th particle, respectively. $V_{i}^{n+1}$ and $x_{i}^{n+1}$ are the updated velocity and position of the *i*th particle at the iteration of *n* + 1, respectively. *c*_1_ and *c*_2_ are learning factors, *r*_1_ and *r*_2_ are two random numbers, ranging from 0 to 1. The process of the PSO algorithm is displayed in Figure 2.

### 2.4. The PSO-SVM Model

**Figure 3:**
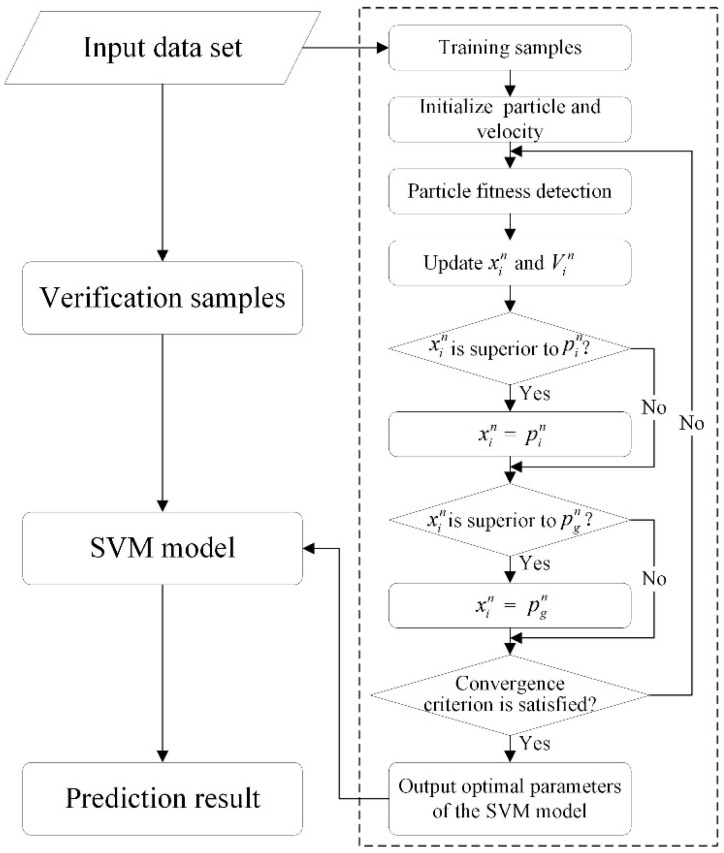
The flowchart of the PSO-SVM model.

In order to improve the performance of the SVM model, the key issue is the selection of the parameters. Although the introduction of a kernel function can achieve the same purpose, the problem of selecting parameters of a kernel function still exists [22]. Combination of the PSO algorithm and SVM model can effectively solve this problem. Taking the RBF function as the kernel function, we demonstrate the flowchart of the PSO-SVM algorithm in Figure 3. To make this algorithm clearer, the details of this algorithm is briefly introduced in Table 1 as follows [22,49]:

## 3. The Proposed GWR-PSO-SVM Model

**Figure 4:**
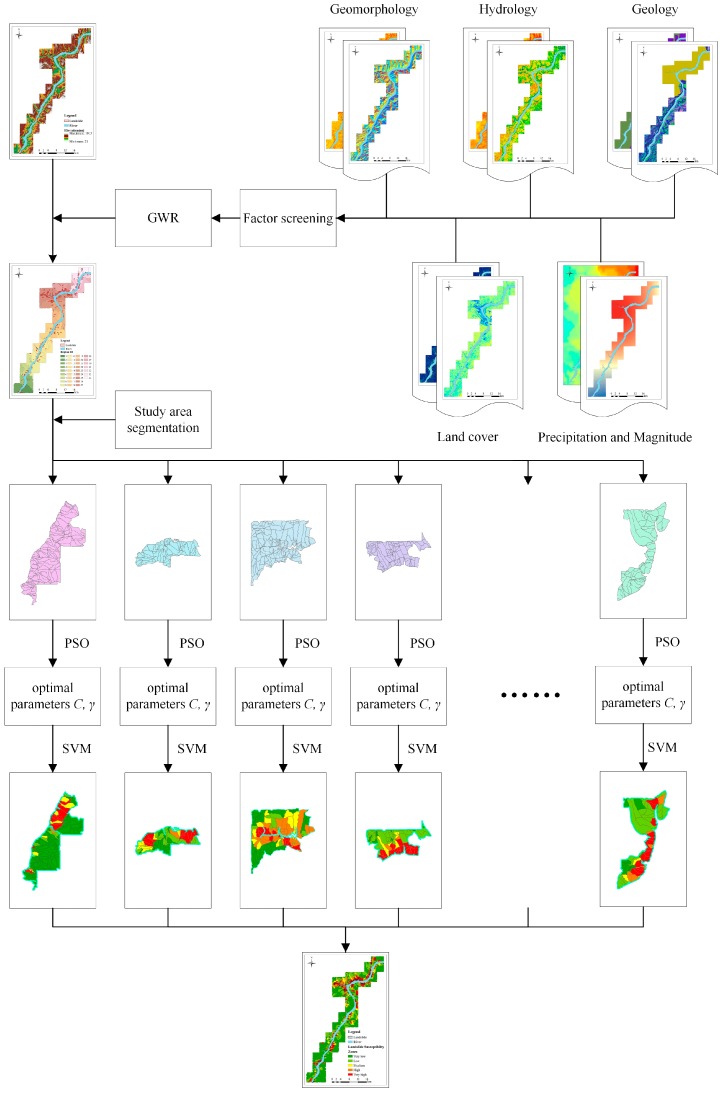
Flow-chart of our proposed method.

In this work, we present a coupled model by combining the techniques of GWR, PSO and SVM. The flowchart of our method is summarized in Figure 4. In the following, each step of our method is briefly introduced.

### 3.1. Factor Screening

It is well-known that some environmental factors have very high correlations. If our coupling model is constructed by using these factors, it may cause errors and cannot effectively improve prediction accuracies. Therefore, it is necessary to screen environmental factors. Correlation analysis is one of commonly used methods for the selection of environmental factors and is considered in our method. In addition, the required environmental factors are further screened based on their importance values. Finally, the remaining environmental factors are used for the subsequent landslide prediction.

### 3.2. Study Area Segmentation

It is well-known that GWR allows different relationships to exist at different points in the study area and improves the modeling performance by reducing spatial autocorrelations [50]. Based on Tobler’s theory about nearness and similarity, observations which are nearer a certain location should have a greater weight in the estimation than observations which are further away [51]. Therefore, we can utilize this technique to estimate parameters for a model at some locations. To segment the study area, we produce and map GWR coefficient values to explore the spatial variability of relationships between the study area and the environmental factors.

The natural breaks method is a typical classification method, which is based on the inherent nature of the packet data [52]. Meanwhile, GWR coefficient values can be used to characterize the spatial autocorrelation of factors. Therefore, we prefer to cluster the study area into several classes in which the GWR coefficient values are greatly similar, with respect to each environmental factor. Meanwhile, it should be noted that the total class number makes great impact on the resultant segmentation maps. Specifically, if the value of *N* is very large, there are too many small partitions in the segmentation map, which causes the difficulties of constructing samples for training and verification and obtaining satisfactory prediction accuracies, as discussed in Section 6.3. In addition, spatial dependency cannot effectively reduced since the region centers are very close. Otherwise, if the value of *N* is too small, there are very few large partitions in the segmentation map, which means that spatial autocorrelations cannot be effectively alleviated in each region and greatly influence prediction results. Furthermore, our method cannot achieve regional scale landslide prediction due to very few prediction regions in the entire study area. To make it clearer, the influence of prediction regions is detailed discussed in Section 6.2.

**Figure 5:**
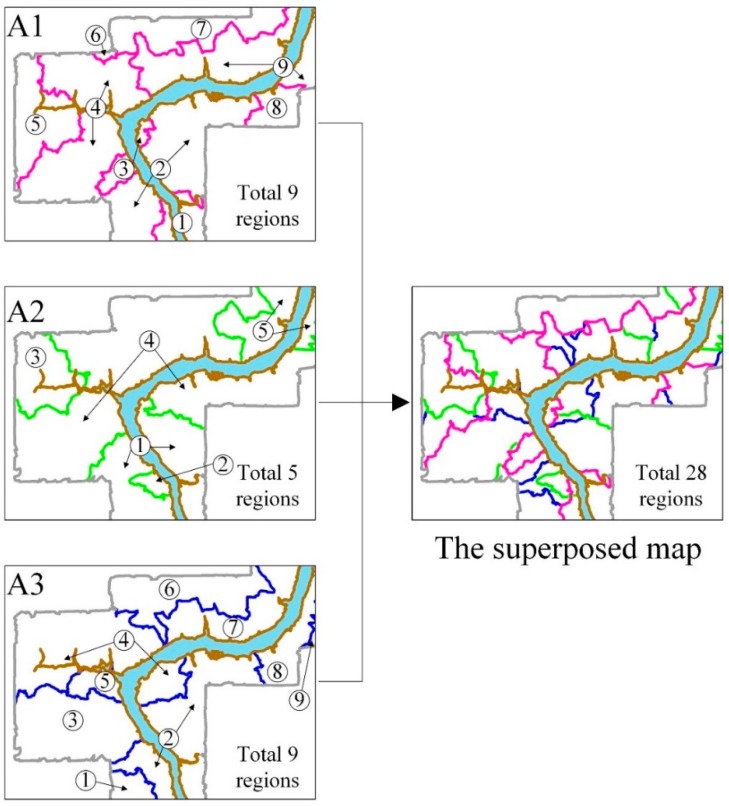
The superposition of classification maps of three environmental factors A1, A2 and A3.

**Figure 6:**
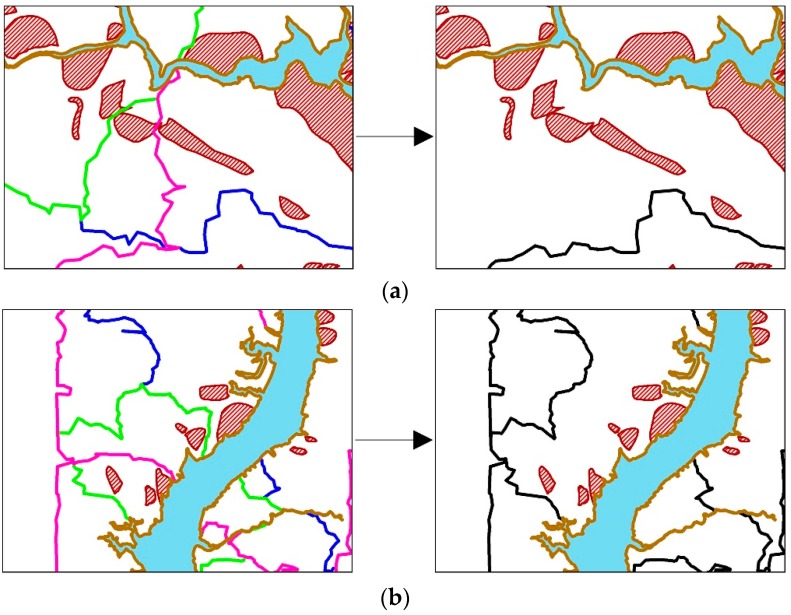

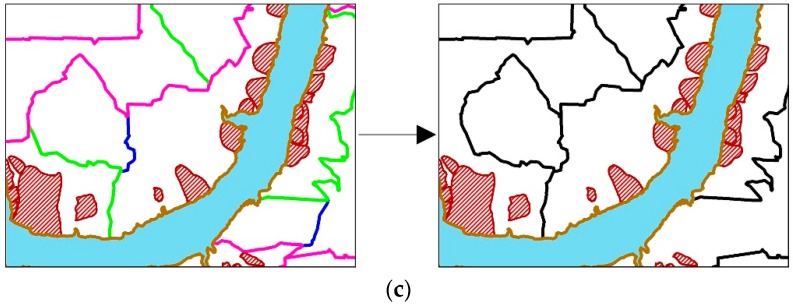
The merging of prediction regions. (**a**) The merging of prediction regions which separate landslides; (**b**) the merging of adjacent small regions including landslides, which are far from other landslides area; (**c**) the preservation of large regions without landslide.

To further weaken spatial autocorrelations, we prefer to superpose classification maps of the selected environmental factors, as shown in Figure 5. Meanwhile, the required environmental factors can be chosen according to importance values of all the environmental factors, measured by the SVM model. It can be observed that the superposition process is a simple intersection of all classes obtained from the most important environmental factors. In addition, the process always results in over-segmentation of the study area, though the GWR coefficient values in each region are consistent for individual environmental factor. As a result, spatial autocorrelations cannot be thoroughly removed since the Euclidean distance between a pair of prediction region centers is too close. In addition, it is very difficult to select training and verification samples for landslide prediction due to quite small regions in the study area. Therefore, it is necessary to merge these small regions in the superposed map. For this aim, the distribution of landslides in the study area should be considered, *i.e.*, (i) prediction regions which separate landslides should be merged as one prediction region; (ii) adjacent small regions including landslides, which are far from other landslides area, should be merged into one prediction region; (iii) a large region without landslide should not be merged with regions containing landslide, as shown in Figure 6.

### 3.3. The GWR-PSO-SVM Model

Once the study area is divided into several prediction regions by clustering GWR coefficients, the SVM model with the kernel function of RBF is used as the prediction component of the coupling model. Moreover, to improve the performance of prediction, the PSO algorithm is embedded into the SVM model to obtain the optimal parameters *C* and *γ* for each prediction region. The details of the GWR-PSO-SVM model for landslide prediction are shown in Table 2 as follows:

## 4. Study Area and Data

### 4.1. General Characteristics

**Figure 7:**
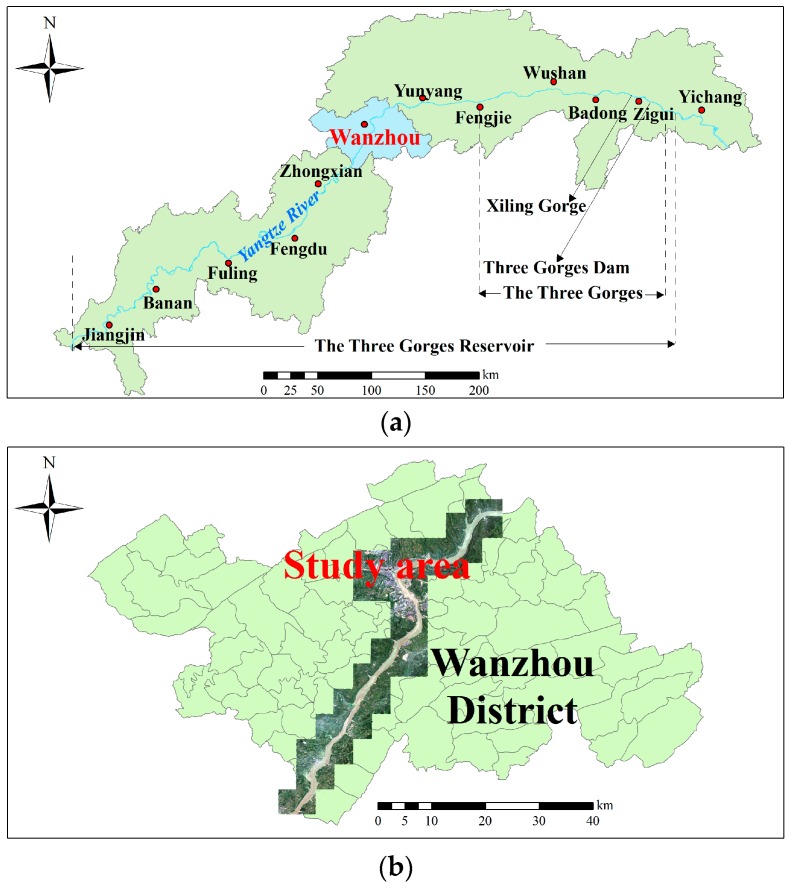

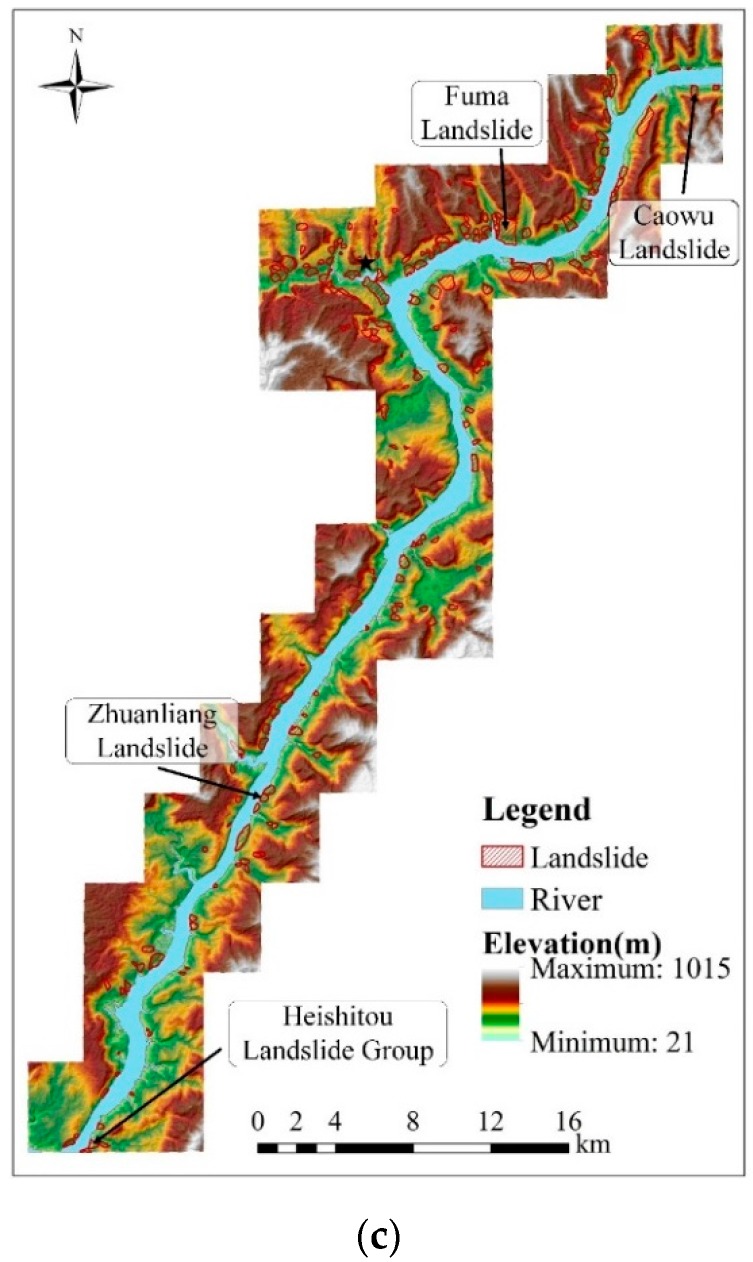
Location map of the study area. (**a**) Site map of the Three Gorges Reservoir; (**b**) site map of our study area; (**c**) digital elevation mode (DEM) overlaid with previously investigated landslides. The red hatched regions represent previously investigated landslides in the study area.

The Three Gorges span from the western Sichuan Basin upstream to the eastern Jianghan Basin downstream [53]. Wanzhou is a district of Chongqing Municipality, bordering Sichuan Province to the northwest and Hubei Province to the southeast. It is one of the main ports of the Yangtze River basin and the important industrial, cultural, trade and transportation center in Yudong. The site covers an area of 3457 km^2^ and lies between longitudes of 107°52’22”–108°53’25” and latitudes of 30°24’25”–31°14’58”, belonging to the subtropical moist climate zone, with a mild climate and abundant rainfall. The annual average precipitation is 1191.3 mm and around 70% of the annual precipitation falls from May to September. Our study area is located in the center of Wanzhou district, distributed along the 80 km-long Yangtze River, with an area of 552 km^2^ and its elevation is between 21 m and 1015 m, as shown in Figure 7.

### 4.2. Geological Setting

**Figure 8:**
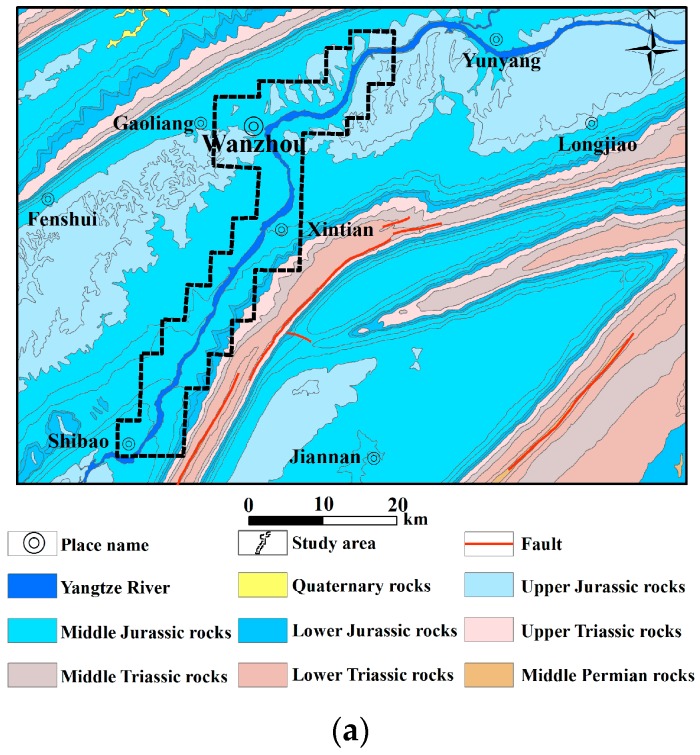

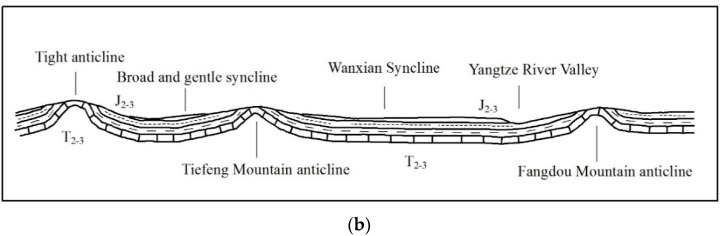
Geological maps of the study area. (**a**) Geological and tectonic sketch; (**b**) a schematic geological cross-section.

The Wanzhou district is located at the two wings of the Wanxian synclinorium of the Eastern Sichuan fold belt. Meanwhile, anticline and syncline exist alternately in this area and construct a typical ejective fold structure [54]. The geological and tectonic framework map and a schematic geologic cross-section of the study area are shown in Figure 8a,b, respectively [55].

### 4.3. Description of Landslides

In the study area, the accurate sizes and shapes of previously investigated landslides can be extracted from the Headquarters of Prevention and Control of Geo-Hazards in Area of Three Gorges Reservoir [56]. In addition, high-resolution aerial photographs are used to detect neogenic landslides which are caused by the impoundment of the Three Gorges Project from 2003, while historical and literature data are employed to identify previous landslides, which were activated during Holocene and/or Pleistocene age, before the impoundment of the Three Gorges Project. In this work, 233 landslides were mapped in the study area.

Note that we cannot obtain terrain data under the Yangtze River, since there are no such information recorded in topographic maps or Advanced Spaceborne Thermal Emission and Reflection Radiometer (ASTER) G-DEM data. As a result, DEM values always vary greatly at the junction between both sides and surface of the Yangtze River, which influences the environmental factors produced by the DEM data. Therefore, we excluded the Yangtze River from the study area. For prediction, computing units are automatically obtained from high-quality digital terrain models (DTMs) by the slope-units method, which is used to partition the territory into hydrological regions between drainage and divide lines [57]. In this work, our study area is divided into 1909 slope-units, including 416 for landslides with total areas of 24.06 km^2^, covering 4.36% of the study area. It can be observed from Figure 7c that the sizes of landslides in this area are very different. For instance, the Fuma landslide with an area of approximately 1.12 km^2^ is the biggest landslide, while the smallest Xianjia 6 group landslide has an area of 3539.77 m^2^.

### 4.4. Environmental Factors of Landslides

In this work, ancillary data used for extraction of environmental factors are the following:

- High-resolution aerial photographs;
- 1:50,000-Scale geological maps [55];
- ASTER G-DEM data with a spatial resolution of 30 m;
- Landsat-8 OLI+ sensor data, acquired on 24 February 2013, with the Path/Row number of 127/39 and its spatial resolution of 30 m for the extraction of land-use and calculation of Normalized Difference Vegetable Index (NDVI) and Normalized Difference Water Index (NDWI);
- Precipitation and seismic data from the China Meteorological Administration and the China Earthquake Administration for obtaining the precipitation and seismic factors.

**Figure 9:**
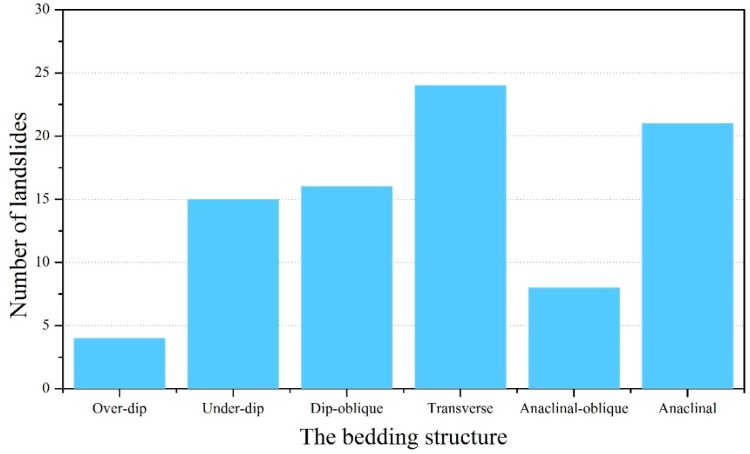
Statistical results of the numbers of landslides corresponding to different bedding structures.

Many researchers have verified the correlations between various environmental factors and landslide occurrence [58]. Based on these contributions and the characteristics of the study area, 29 environmental factors are selected to predict the potential distribution of landslides, including geomorphological, geological, hydrological, land cover, meteorological and geophysical factors. The selected environmental factors and their original values are listed in Table 1. In particular, the classification for the bedding structure is shown in Table 3. This factor is based on the topography bedding intersection angle (TOBIA) index [59] using the slope aspect, slope angle, bed dip direction and bed dip angle in space. In addition, the numbers of landslides corresponding to different bedding structures are demonstrated in Figure 9. From this figure, landslide failure can be caused by any type of slope in Figure 9. It should be mentioned that there are many horizontal strata landslides in the study area [60]. Since the formation mechanism of this type of landslides is very complicated and beyond the scope of this article, the gently dipping structure is not addressed in this work. Meanwhile, the figure depicts that there are very strong relationships between the different types of slope and the occurrence of landslides. Therefore, this factor is an important indicator of landslide and should be taken into account for prediction.

It is known that the slope-units method is different from the grid-cells one, because the former is irregular, which means that the resultant areas by the slope-units method are different from each other. Therefore, the first problem of the slope-units method is that how to assign a normalized value to each slope-unit. If the original value of an environmental factor in Table 4 is a continuous variable, such as elevation, slope angle, terrain surface convexity and so on, the mean value of this factor is computed as the normalized value of the corresponding slope-unit, while if the original value of an environmental factor is a discrete variable, such as slope form, lithology, bedding structure and land-use, the most frequently occurring value of this factor is used as the value of this slope-unit. By using this idea, the 1909 slope-units are assigned to a unique value of each factor. To obtain landslide susceptibility of the study area, this value is used in all prediction models in this work.

## 5. Results

### 5.1. Experimental Results of The GWR-PSO-SVM Model

**Figure 10:**
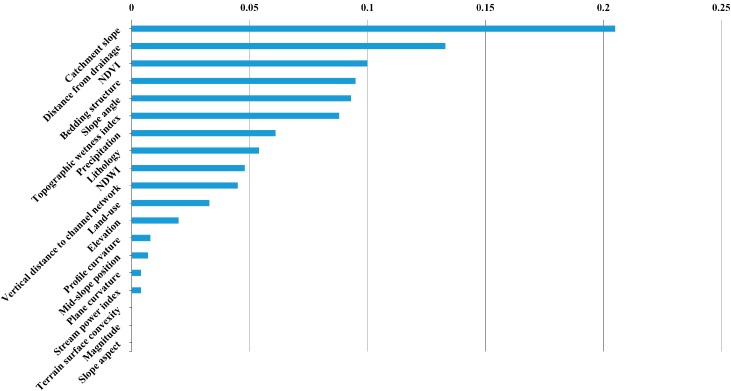
Importance values of the remaining 19 environmental factors.

As mentioned in Section 3.3, the classical PPMCC is used to weaken the correlations of the selected environmental factors and *T*_1_ = 0.5. For simplicity, correlations of geomorphological and hydrological factors are listed in Table 5 and Table 6 and 10 factors are excluded for all the models used here. As a result, the remaining 19 environmental factors are relatively independent and can be further screened based on their importance values ranging from 0 to 0.205, as illustrated in Figure 10, obtained using SPSS Clementine 12 software (IBM, Armonk, NY, USA). To this end, we set *T*_2_ = 0.02 and exclude the environmental factor whose importance value is less than *T*_2_. Finally, 12 environmental factors are selected for the construction of the coupling model, *i.e.*, catchment slope, distance from drainage, NDVI, bedding structure, slope angle, topographic wetness index, precipitation, lithology, NDWI, vertical distance to channel network, land-use and elevation.

**Figure 11:**
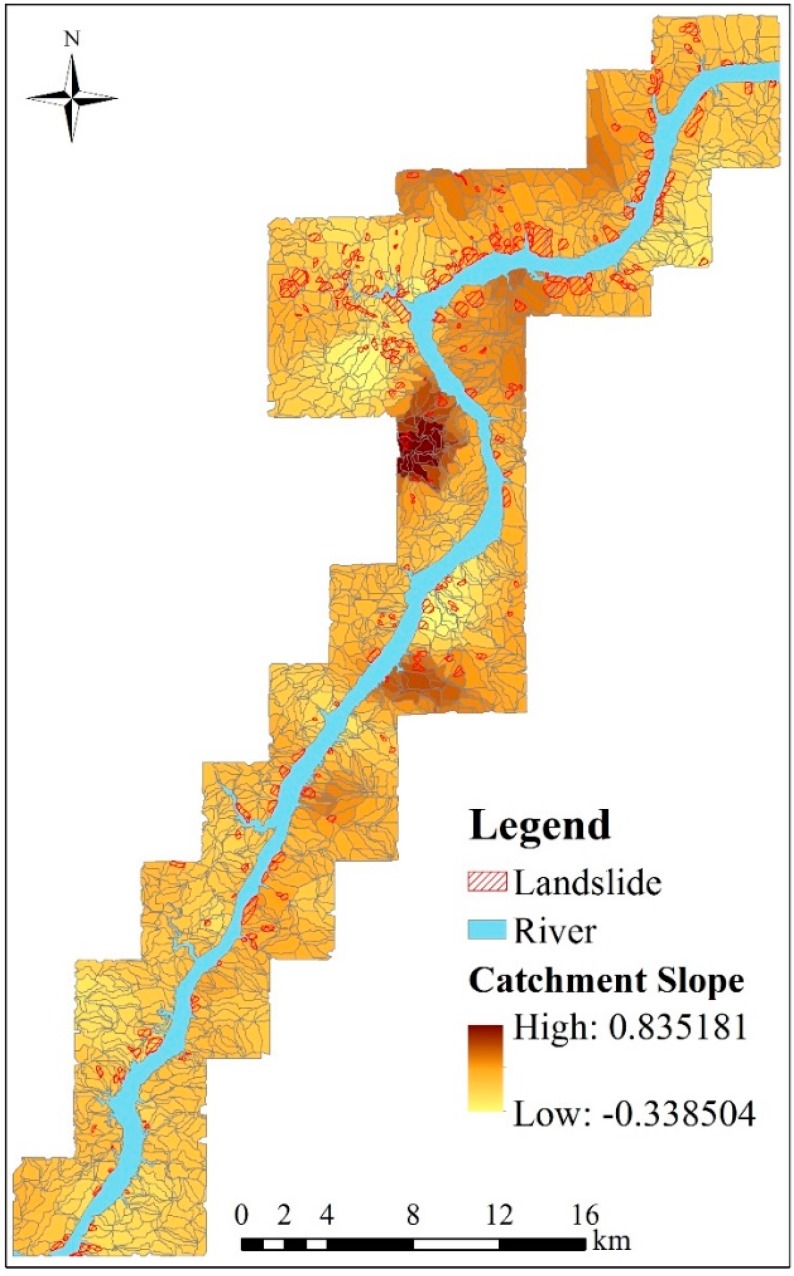
The GWR coefficient map of the catchment slope.

According to the selection criterion mentioned in Section 3.2, the most important environmental factors, *i.e.*, catchment slope, distance from drainage and NDVI, are selected as the regional division factors, whose GWR coefficients are obtained by exploiting an adaptive bi-square kernel and AIC in the GWR method. The GWR coefficient values of catchment slope are shown in Figure 11. It can be easily observed from the figure that different clusters with respect to GWR are spatially developed. Based on the relationship between GWR and spatial autocorrelation mentioned in Section 1, we can easily infer that the GWR coefficients in each cluster are very close. Consequently, spatial dependency are greatly reduced if each cluster is considered as a spatial variable. Therefore, it is possible that the study area can be partitioned into different prediction regions while spatial autocorrelations are very limited.

**Figure 12:**
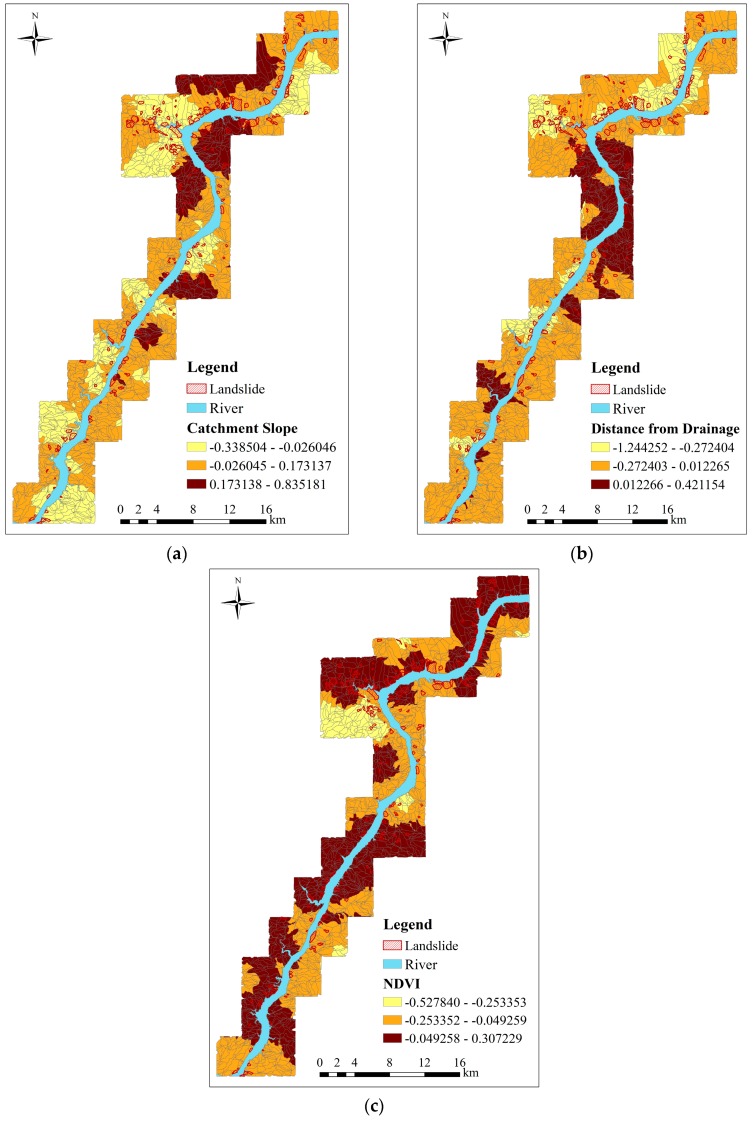
The GWR coefficient values and classification maps of environmental factors. (**a**) Catchment Slope; (**b**) the distance from drainage; (**c**) NDVI.

**Figure 13:**
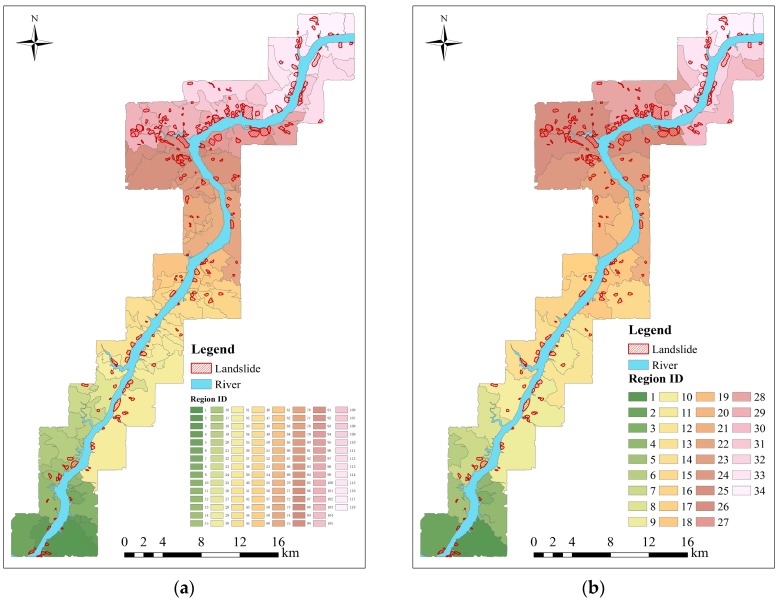
The resultant prediction regions of the study area. (**a**) The resultant prediction region map after the superposition process; (**b**) the final prediction region map after the merging process.

In this work, we set *N* = 3, *i.e.*, these selected environmental factors are clustered into three classes by the natural breaks method and the corresponding classification maps are shown in Figure 12a–c. For convenience, the slope-unit without landslide is named as the non-landslide slope-unit, while the slope-unit including landslide is named as the landslide slope-unit. The result of simple superposition is shown in Figure 13a. According to the three rules for merging regions mentioned in Section 3.2, the study area is finally divided into 34 prediction regions by superposing all classification maps. For simplicity, each prediction region is assigned to a unique label, as shown in Figure 13b. It can be observed from this figure that 25 regions contain landslides in the study area. The numbers of the slope-units and the landslide slope-unit are listed in Table 7.

**Figure 14:**
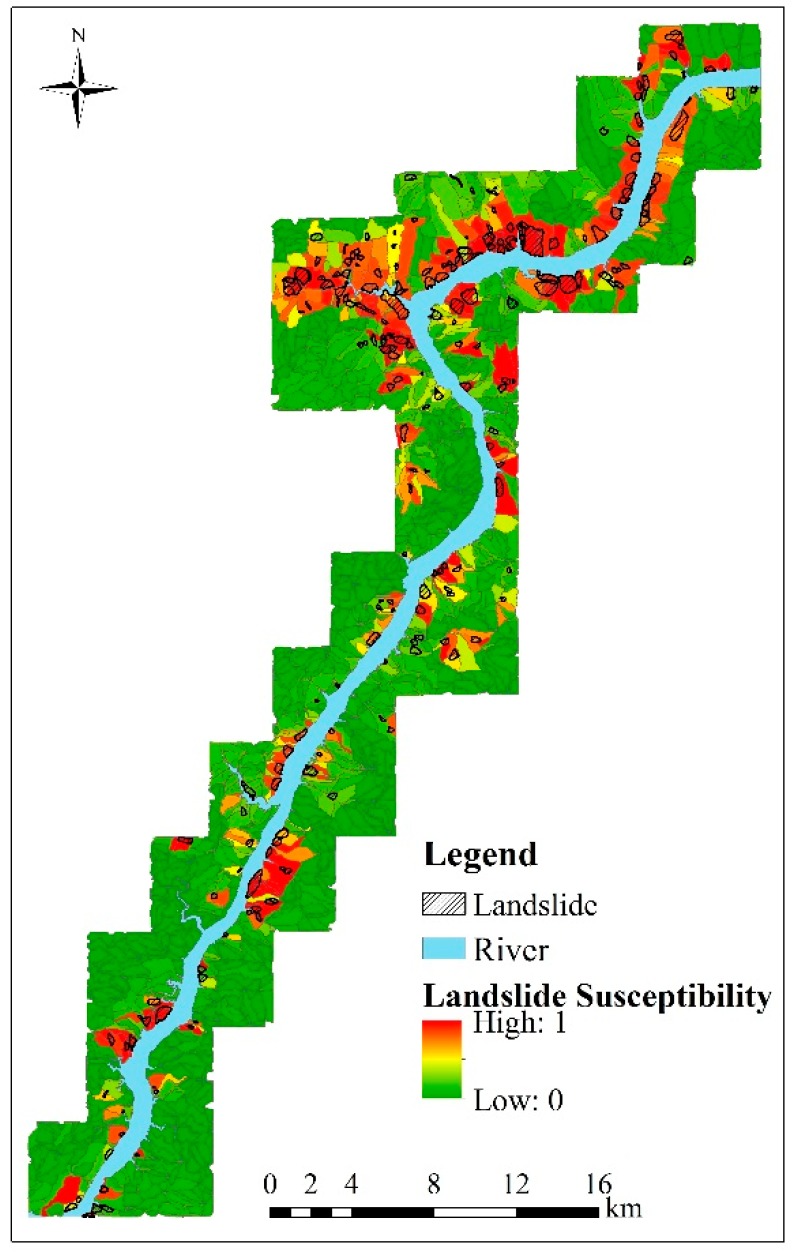
The landslide susceptibility map by the GWR-PSO-SVM model.

For the GWR-PSO-SVM prediction model, all of prediction regions must be sampled as input variables. For each prediction region in Figure 13b, the label of the landslide slope-unit is assigned as “1”, while the label of the non-landslide slope-unit is assigned as “0”. In our experiment, we use the same number of landslide slope-units and non-landslide slope-units in each prediction region to form training and verification samples. It can be observed from Figure 13 that the total number of non-landslide slope-units in each prediction region is always more than that of the landslide slope-units. Therefore, all of the landslide slope-units and the same number of the randomly selected non-landslide slope-units form the required samples. Meanwhile, the proposed GWR-PSO-SVM model is a local model, which generates the optimal *C* and *γ* of the SVM model for each prediction region by using the PSO algorithm, as shown in Table 8. It should be noted that the prediction regions without landslides are not included in this table. Meanwhile, we perform the SVM classifier to estimate the likelihood that each slope-unit contains the existing landslides and demonstrate the corresponding probability maps in Figure 14. The probability value in the map ranging from 0% to 100% represents the different degrees of landslide susceptibility.

### 5.2. Methods to Assess Models Performance

To objectively evaluate the performance of the models considered, three methods are utilized. The first measure is overall prediction accuracy, which is used to evaluate prediction correctness and can be defined as:

(11) $$p=\frac{a+b}{S}\cdot 100\%$$

where *a* and *b* are the numbers of correctly predicted landslide and non-landslide slope-units in the landslide susceptibility maps, respectively. *S* is the total number of slope-units in the study area. According to (11), this measure can be appropriately applied to evaluate the global models, such as the SVM, PSO-SVM, RS-SVM models, by taking into account the entire study area. While it is used for the GWR-based models, the measure can be computed in each prediction region. In this work, the final measure of overall prediction accuracy is defined as follows:

(12) $$p=\frac{\sum_{i=1}^{n_{pr}}\left(a_{i}+b_{i}\right)}{\sum_{i=1}^{n_{pr}}S_{i}}\cdot 100\%$$

where *i* = 1,2,…,*n_pr_* (*n_pr_* is total number of prediction regions), *a_i_* and *b_i_* are the numbers of correctly predicted landslide and non-landslide slope-units in the *i*th prediction region, respectively. *S_i_* is the number of slope-units involved in the current prediction region.

The second measure is exploited to evaluate prediction accuracy of landslide areas in each class of landslide susceptibility maps obtained by the mentioned models according to the distribution of our study area. This measure is named as class-specific accuracy and is defined as follows:

(13) $$p_{j}=\frac{A_{j}}{B_{j}}\cdot 100\%$$

where *j* = 1,2,⋯,*M* (*M* is total number of landslide susceptibility zones), *A_j_* and *B_j_* are the numbers of landslide slope-units and total slope-units in the *j*th landslide susceptibility zone, respectively. To perform this measure, our study area is classified into *M* landslide susceptibility zones. In this work, the fixed interval method is used to achieve this aim and it is based on previous studies to segment study areas by the predefined thresholds, which is widely used for comparison of multiple models [7,46,61].

The third measure is the classical receiver operation characteristic (ROC) curve and its area under curve (AUC). In a ROC curve the true positive rate (sensitivity) is plotted in function of the false positive rate (100-specificity) for different cut-off points. Each point on the ROC curve represents a sensitivity/specificity pair corresponding to a particular decision threshold. A test with perfect discrimination (no overlap in the two distributions) has a ROC curve that passes through the upper left corner (100% sensitivity, 100% specificity). Therefore, the closer a curve is to the upper left corner, the better are the prediction results [62].

### 5.3. Comparison with Further Models

To better demonstrate the performance of our model, several models are compared to our method, including: (1) the SVM model, in which the study area are globally used for sampling and prediction; (2) the PSO-SVM model, in which the PSO algorithm is used to obtain the optimal *C* and *γ* to improve prediction accuracies; (3) the landslide susceptibility mapping method based on rough set (RS) and SVM proposed by Peng *et al.* [46]. RS theory is an effective tool introduced by Pawlak [63] and discussed in many review papers [64,65,66,67,68,69,70]. This technique can deal with vagueness and uncertainty information and identify cause-effect relationships in databases as a form of data mining and knowledge discovery [46,63,71]. Meanwhile, it has been widely used in various disciplines of science [72], including remote sensing [73], geographic information science [74], and landslide susceptibility mapping [71], *etc.* In the work of [46], it was employed to select key environmental factors for landslide prediction; (4) the GWR-SVM model, which is a local model and similar to our coupling model, without the PSO step to obtain the optimal *C* and *γ*.

For a fair comparison, the same mapping unit and original environmental factors are used for all models used here. It should be noted that the RS-SVM model is different from the other models due to the fact that its input environmental factors are determined based on the RS theory after the PPMCC analysis. In our experiments, all of the remaining 12 factors are used for input variables for the SVM, PSO-SVM, GWR-SVM and our models, while 14 factors are selected based on the RS theory in the RS-SVM model, excluding land-use, mid-slope position, plane curvature, stream power index, terrain surface convexity from the remaining 19 factors.

It is well-known that the selection of samples for training and verification is a key step for the SVM prediction model. As mentioned above, the classical SVM, PSO-SVM and RS-SVM models can be considered as global ones due to the fact that the entire study area is taken into account for selecting samples, *i.e.*, all of the landslide slope-units in the study area and the same number of the randomly selected non-landslide slope-units are used for training their respective SVM models, while all of the slope-units in the study area are utilized for verification. Nevertheless, the selection scheme of the remaining GWR-based models is performed for each prediction region, instead of the entire study area, as mentioned in Section 5.1. Therefore, the sample size of each model in this work is measured using the number of slope-units in the study area or each prediction region. Table 9 depicts the training and verification sample sizes of all the models. In addition, the PSO algorithm is used for the PSO-SVM and GWR-PSO-SVM models to obtain the optimal *C* and *γ* to improve prediction performance of the SVM model.

**Figure 15:**
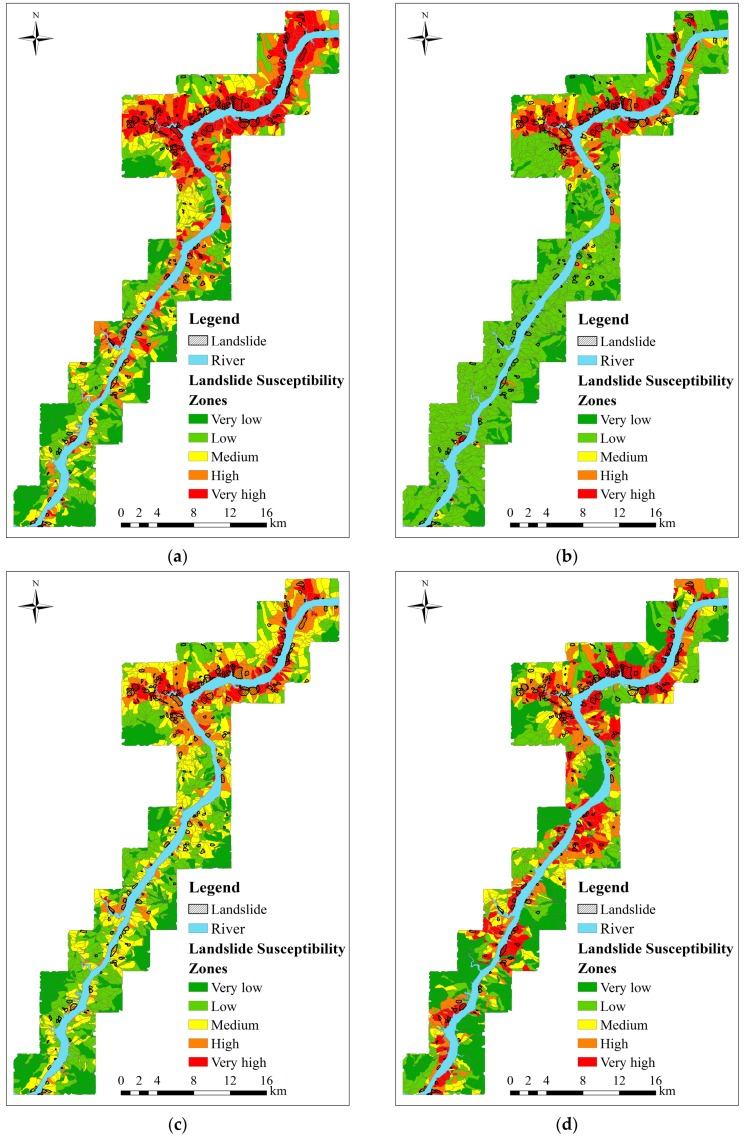

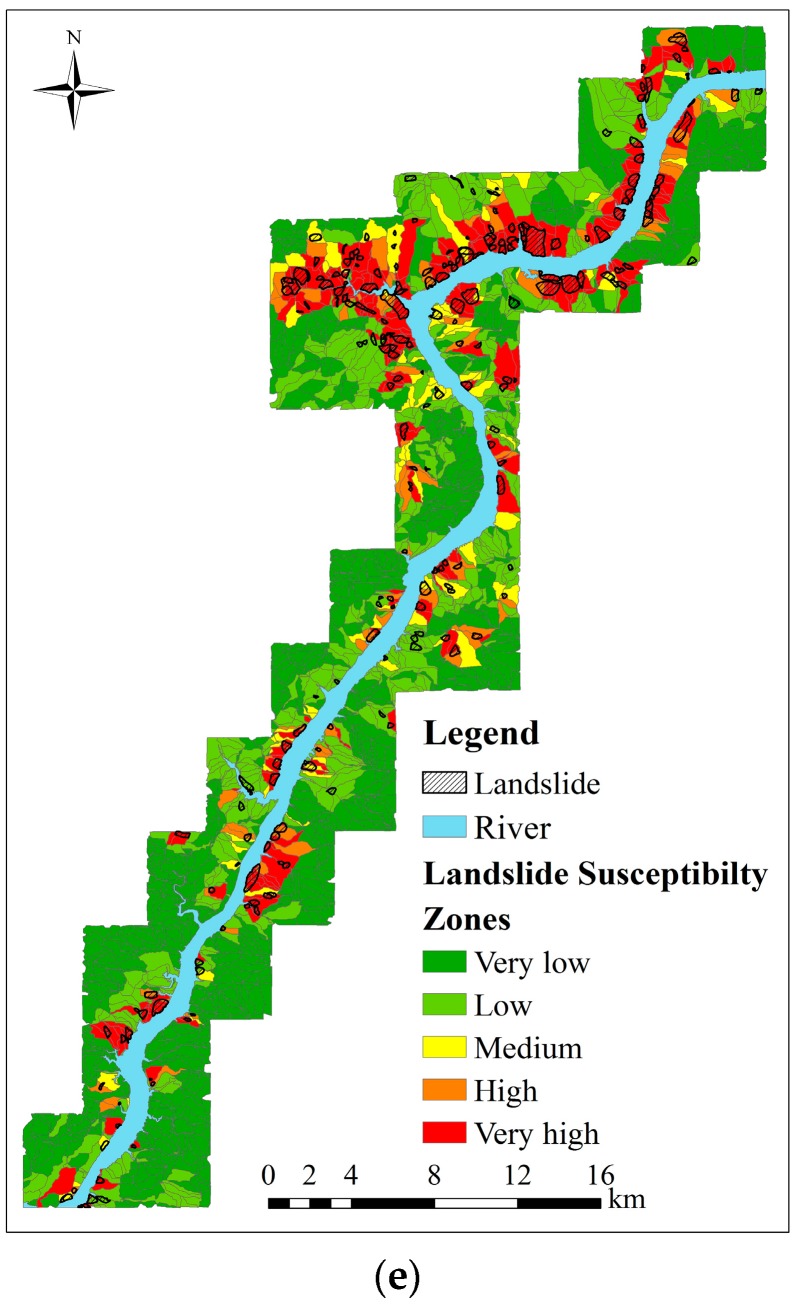
Landslide susceptibility zoning using the fixed interval method. (**a**) SVM; (**b**) PSO-SVM; (**c**) RS-SVM; (**d**) GWR-SVM; (**e**) GWR-PSO-SVM.

To make probability maps more readable, we can divide probability values by using fixed interval method in ArcGIS software into five susceptibility categories, *i.e.*, very low, low, medium, high and very high, corresponding thresholds are fixed to 0.1, 0.35, 0.75 and 0.9, respectively, as shown in Figure 15. It can be observed from Figure 15 that all of the models can achieve the purpose of landslide prediction. Meanwhile, the very high-susceptibility zones are apparently mapped in the main urban area of Wanzhou district in all the susceptibility maps, which accords with the fact that the previously investigated landslides are mainly distributed in this area. The distribution of high and very high-susceptibility zones is greatly different for each model.

For instance, most of the previously investigated landslides are located in high or very high-susceptibility zones in the maps of the SVM, RS-SVM and GWR-SVM models. However, a large number of slope-units are unreliably classified by these models as high or very high-susceptibility zones as well. Landslides are typically a minority class in the study area, the PSO algorithm always results in local optima of the SVM model, when it is applied to the entire study area. As a consequence, the previously investigated landslides in the southwest of the study area cannot effectively be predicted by the PSO-SVM model. In contrast, the map by our model is consistent to the ground truth of landslide distribution. Although the PSO algorithm is used in our method to optimize the parameters in the SVM model, the division of our study area into prediction regions with appropriate sizes can greatly overcome trapping in local optimum. The high and very high-susceptibility zones mainly concentrate in the previously investigated landslide areas, while most of non-landslide areas are classified as low and very low-susceptibility zones, which guarantee the reliability of prediction results of landslide susceptibility. The overall accuracies of landslide susceptibility mapping by all the models used here are listed in Table 10.

**Figure 16:**
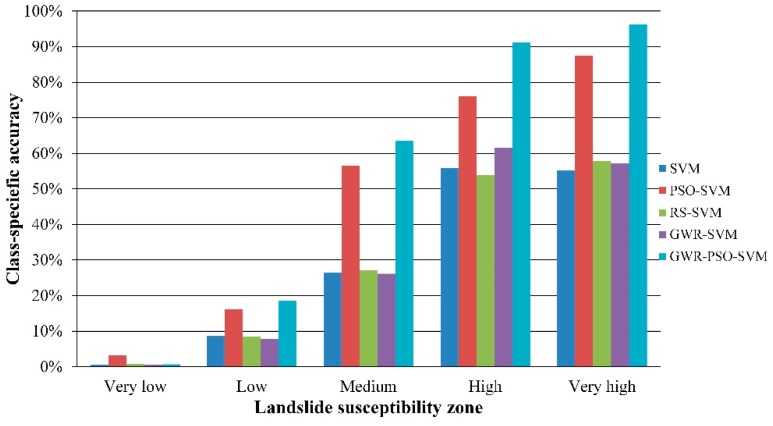
The class-specific accuracies by different prediction models using the fixed interval method.

In this table, the item of “Correct” indicates the number of slope-units that are correctly predicted in prediction regions, while the item of “Total” means the number of slope-units in prediction regions. It should be noted that this “total” number in the GWR-SVM and GWR-PSO-SVM models are calculated using the prediction regions including landslides. It is obvious that the GWR-PSO-SVM model can achieve the best prediction accuracy of 91.10%, which is 7.8%–19.1% higher than the traditional SVM-based models. To further compare the performance of all the models, the class-specific accuracies are shown in Figure 16. It can be clearly seen that the class-specific accuracy of the very high-susceptibility zone achieved by our model is highest (96.27%) when compared with the other models, which means that our model can detect the very high-susceptibility zones mainly including the previously investigated landslides.

**Figure 17:**
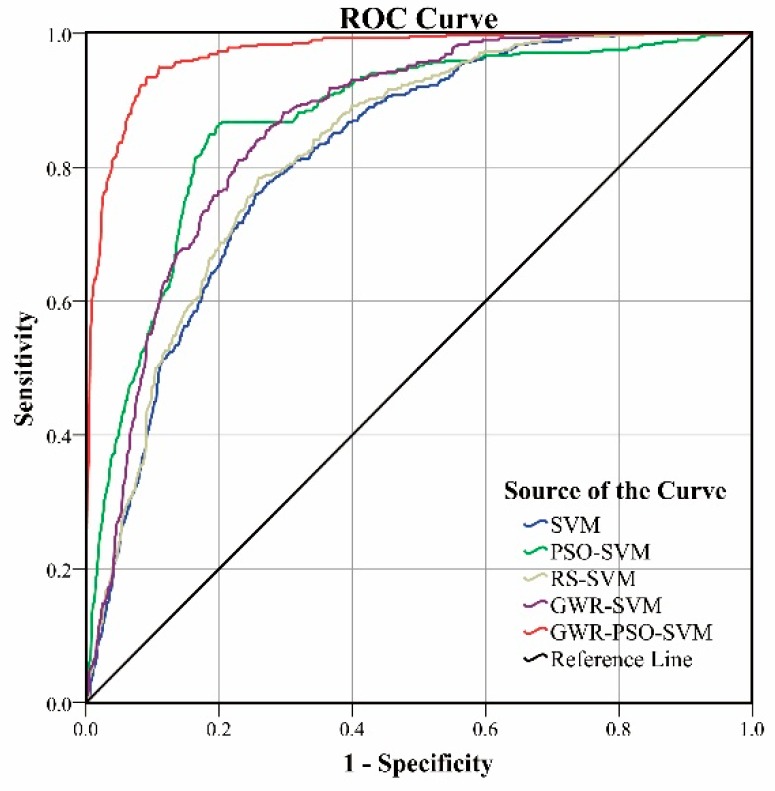
The ROC curve of all the prediction models.

The ROC curves of all the methods are plotted in Figure 17. It is known that the closer the ROC curve is to the upper left corner, the higher the overall accuracy of the test is. As can be observed from Figure 17, we can obtain similar conclusions as for the two previous evaluation measures, *i.e.*, the GWR-PSO-SVM model can achieve the best prediction result. Meanwhile, the ROC plots of the GWR-SVM and the RS-SVM models are pretty close to each other. Since the PSO algorithm is not very robust when it is applied to the whole study area, the ROC plot of the PSO-SVM model is not continuous and is close to the upper left corner when the value (of the 1-specificity) is 0.2, but worse than the RS-SVM model, GWR-SVM and our models when the value is larger than 0.2. In addition, the corresponding AUC is listed in Table 11. The larger the value of AUC, the better the performance of the prediction model. As shown in this table, our model can produce the largest area of 0.971, when compared with the other models.

It should be noted that there are a few non-landslide regions in the prediction region map (Figure 12b), since landslides are typically a minority class in the study area. To compare the performance of our model with the global models, we assume in this work that the overall prediction accuracies of these non-landslide regions are 100%, which may improve the overall accuracy of the entire study area. Meanwhile, our experiments not reported here confirm that the AUC value of our model can still reach 0.962 by removing these non-landslide regions from the study area. Furthermore, all the prediction models were applied to Zigui to Badong section in the Three Gorges Reservoir for landslide susceptibility mapping. The experimental results demonstrated that the GWR-PSO-SVM model can obtain the best prediction result as well and the AUC value of 0.965, which is highest among all the models. Therefore, the universality of our model can be validated. Finally, to objectively compare our model with the other models, we select the same number of landslide slope-units and non-landslide slope-units in each prediction region. Although the number of training samples is relatively small in certain prediction regions, the influence on the overall prediction accuracy is very limited.

## 6. Discussion

### 6.1. Impact of Environmental Factors

**Figure 18:**
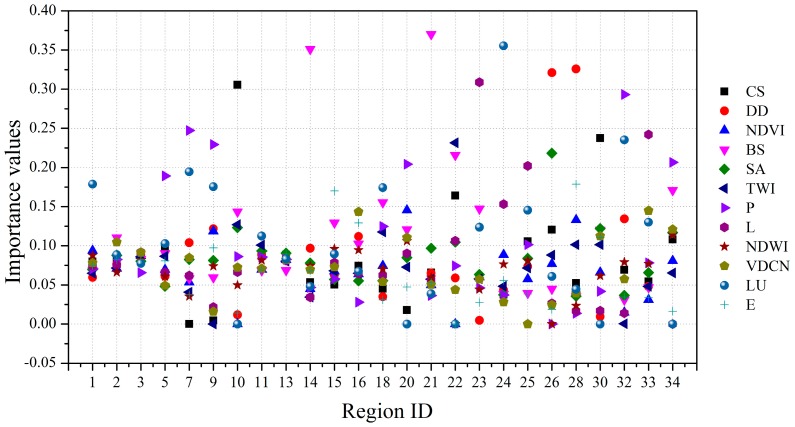
The importance values of the environmental factors in different prediction regions.

It should be noted that the global and regional prediction results of the study area are always different, mainly due to two reasons. The first one is the prediction model. Since the SVM model has been used as a universal model and can obtain satisfactory results, it is exploited by all the models used here for landslide susceptibility mapping. The second one is the impact of environmental factors. There are several crucial environmental factors for landslide prediction, such as elevation, slope angle and so on. However, the most crucial factors are different in different parts of the study area. For instance, the environmental factor of distance from drainage is greatly significant for landslide failures in the area along the Yangtze River, while slope angle may be the most important environmental factor in the areas far away from the Yangtze River. Therefore, the introduction of the GWR technique into landslide susceptibility mapping may avoid these two problems and improve the prediction accuracy. The importance values of all the environmental factors in each prediction region, obtained using SPSS Clementine 12 software, are displayed in Figure 18. It can be observed that the importance values of the final 12 environmental factors produced in Section 5.1 at each prediction region are different. Meanwhile, in each prediction region, the rank of each environmental factor in terms of the important value is greatly different.

### 6.2. Influence of Regions Number

**Figure 19:**
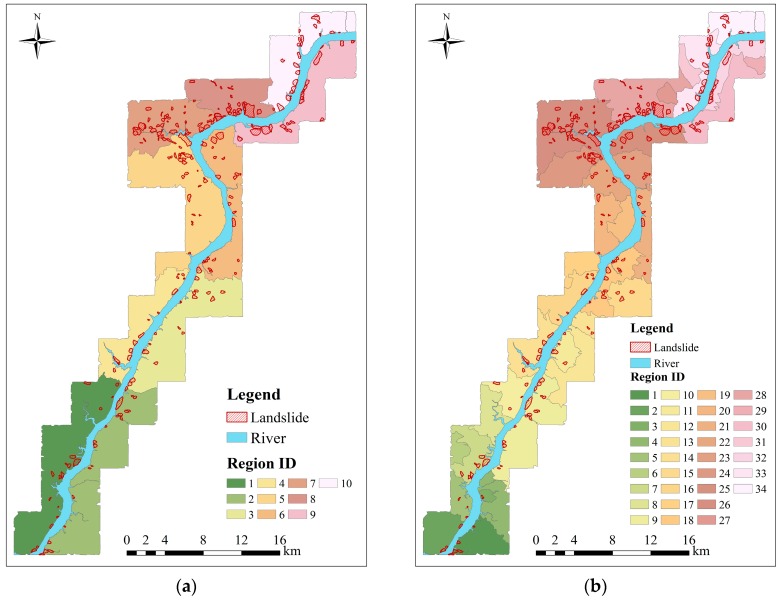

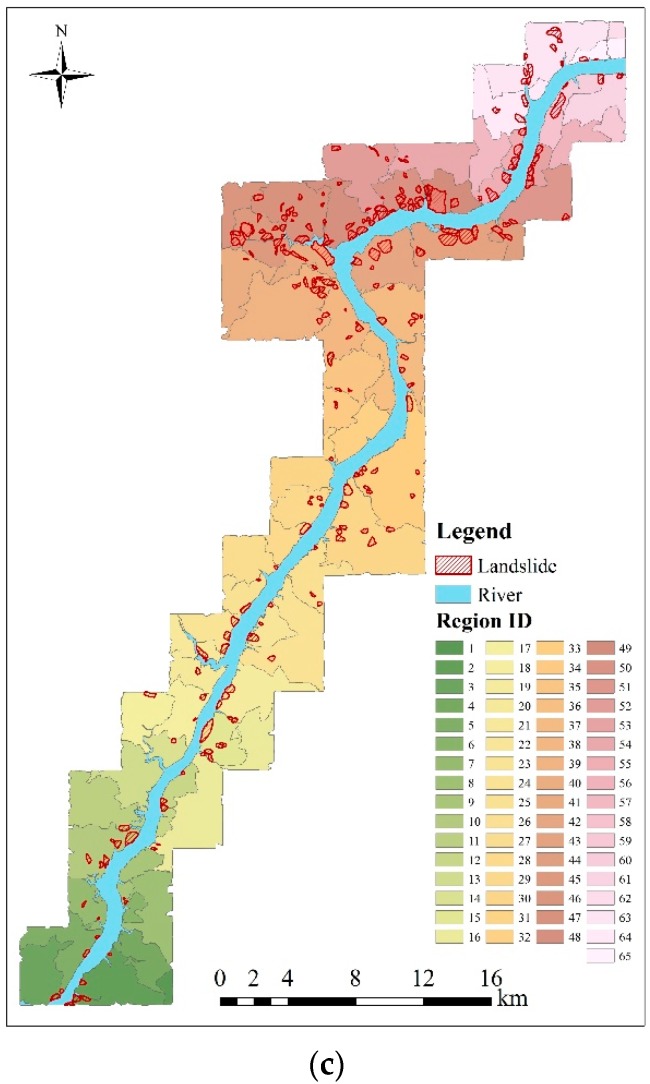
The performance of segmentation with respect to different values of *N*. (**a**) *N* = 2; (**b**) *N* = 3; (**c**) *N* = 4.

To demonstrate the impact of the performance of segmentation of the study area, the resultant segmentation maps, with respect to different values of *N* from 2 to 4, are shown in Figure 19. In Figure 19a, the study area is divided into 10 prediction regions when *N* = 2 which may avoid the problem that the importance rank of each environmental factor is not the same in different prediction regions. However, the impact of each environmental factor in different spatial positions is not taken into account.

For instance, all the prediction regions are produced distributing from the Yangtze River to boundaries of the study area, but the importance rank of each environmental factor may greatly change in different parts of each prediction region, which cannot be carefully considered in prediction models if prediction regions are very large. In Figure 19c, the study area is segmented into 65 prediction regions if *N* = 4. In this way, the slope units may be very few in prediction regions. As a consequence, the landslide and non-landslide slope units in each prediction region are not enough to constitute required samples, which influences landslide prediction accuracies. In contrast, our study area in this work is divided into 34 prediction regions by choosing *N* = 3 and different impacts of environmental factors in these regions are effectively utilized into prediction models. In addition, the size of each prediction region is appropriate for obtaining the required samples, as shown in Figure 19b.

### 6.3. Model Sensitivity

**Figure 20:**
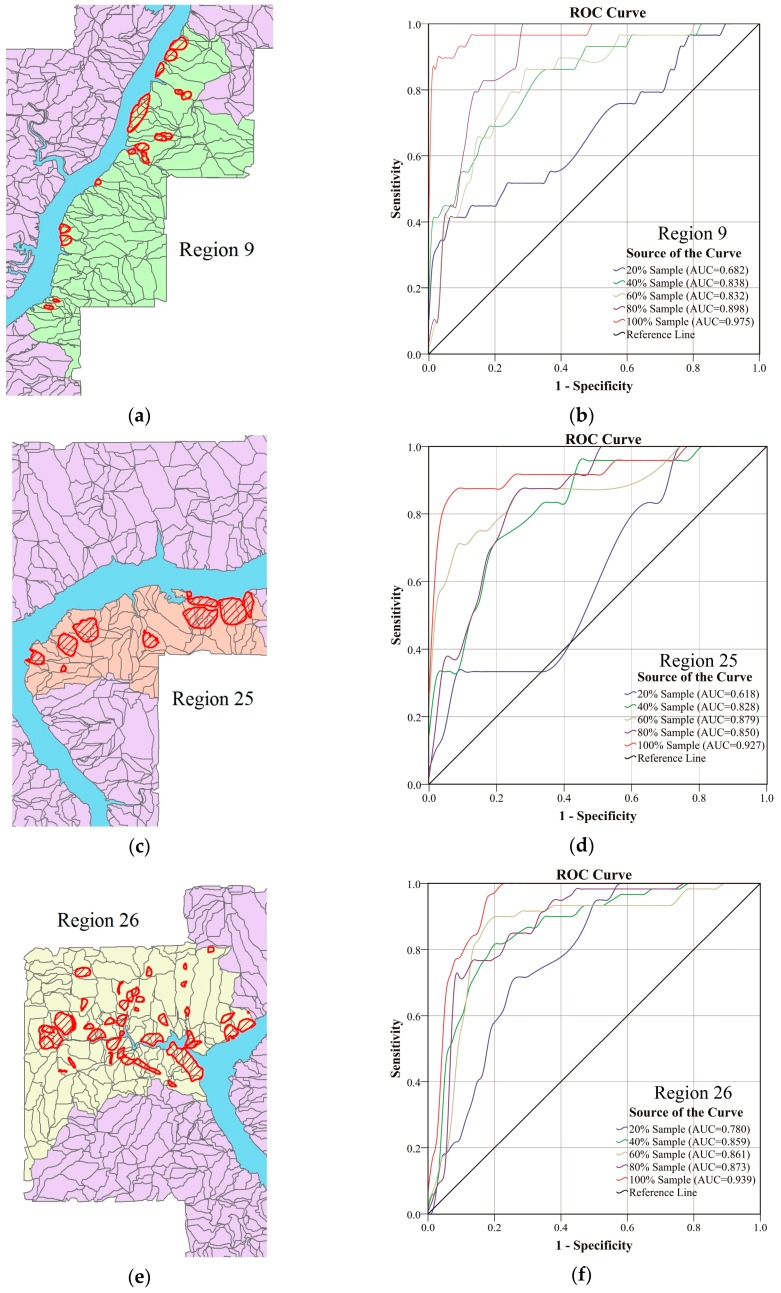

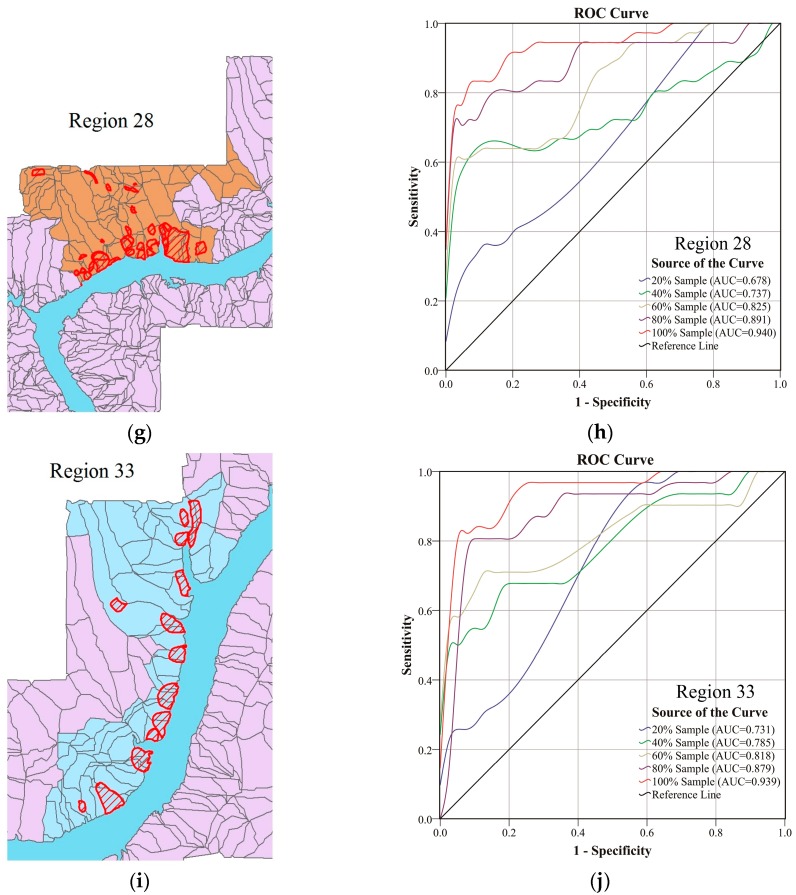
The ROC curves with different sample sets for the five regions. (**a**) The prediction region No. 9; (**b**) the ROC curves and AUCs corresponding to (**a**); (**c**) the prediction region No. 25; (**d**) the ROC curves and AUCs corresponding to (**c**); (**e**) the prediction region No. 26; (**f**) the ROC curves and AUCs corresponding to (**e**); (**g**) the prediction region No. 28; (**h**) the ROC curves and AUCs corresponding to (**g**); (**i**) the prediction region No. 28; (**j**) the ROC curves and AUCs corresponding to (**i**).

To evaluate the sensitivity of the proposed model to the number of prediction and verification samples, five prediction regions, which have the most landslide slope-units, are selected to obtain ROC curves of the prediction performance by choosing five different percentages of required sample sets, *i.e.*, 20%, 40%, 60%, 80% and 100%. The corresponding prediction regions in our study area and their ROC curves are depicted in Figure 20. In general, the higher percentage of the required samples we choose, the better the prediction performance, *i.e.*, the prediction accuracy of our model is highest when using all of the required samples, while it is lowest when only 20% of the required samples are used in our model. The prediction results are greatly determined by the selection of samples due to the complexity of landslides in the study area. If training samples are very small, we cannot extract valuable information from environmental factors, which makes it difficult for our model to guarantee accuracies of landslide prediction. In addition, the selection of the required samples in each prediction region results in fewer training samples for prediction. As a result, the prediction accuracy of our model is lower as the training samples are reduced.

## 7. Conclusions

In this paper, an effective PSO-SVM method based on the GWR technique is presented for landslide susceptibility mapping at a local scale by integrating multisource data of the Wanzhou district in the middle of the Three Gorges Reservoir, China. It has been reported that landslide events occurred in the last three years in the main urban area of the Wanzhou district. In this work, a GWR algorithm is used in our model to segment the study area into a series of prediction regions with appropriate sizes by clustering slope units. Then, a PSO-SVM prediction model is applied to each prediction region for landslide susceptibility mapping. This allows the proposed GWR-PSO-SVM model can obtain accurate landslide susceptibility maps at a regional scale. Experimental results demonstrate that coupling different models as in the GWR-PSO-SVM model can achieve better prediction performance, when compared to the traditional SVM-based models. Meanwhile, these landslide prediction models are comprehensively evaluated using three objective measures including the overall prediction accuracy, the landslide susceptibility class-specific accuracies, and the ROC curves and AUC values. We can draw the following conclusions: (1) The GWR-PSO-SVM model can obtain the best overall accuracy of 91.10%; (2) The GWR-PSO-SVM model can achieve the highest class-specific accuracy of 96.27% with respect to the very high-susceptibility zones, which are mainly covered with the previously investigated landslides; (3) The GWR-PSO-SVM model can achieve a more reliable ROC curve and a higher AUC value of 0.971. Therefore, our model can achieve superior prediction performance to the traditional prediction models. In future, a further improvement can be achieved by selecting more reasonable segmentation factors and performing segmentation postprocessing.

## Figures and Tables

**Table 1:** Procedures of the PSO-SVM algorithm.

**Input: Training and Verification Samples.**
**Output: The Result of the PSO-SVM Model.**
**1.** **Initialization parameters:** Generate initial particles comprised of *C* and *γ* of the SVM model. And set the PSO parameters including number of iterations, population size, maximum iteration number, the learning factors, the initial particle swarm location, the random flight velocity and two random number in range [0,1]. Initial the iteration = 0, and perform the training process from step **3–9** for each particle.
**2.** **Data set:** Selection the training and verification samples.
**3.** Set iteration *n* = *n* + 1.
**4.** **SVM model training:** Conduct 10-fold cross validation (CV) on the training samples, and calculate the average CV accuracy based on the (*C*, *γ*).
**5.** Evaluate its fitness by the average CV accuracy which is obtained in step **4**.
**6.** Update the global and local optimal solution according to the result of the fitness evaluation.
**7.** Each particle moves to its new location $x_{i}^{n}$ by velocity $V_{i}^{n}$ according to Equation (10).
**8.** Until this iteration, the local optimal solution of the *i*th particle $p_{i}^{n}$, are compared with the new location $x_{i}^{n}$, the better will be the new $p_{i}^{n}$ in the iteration *n*+1. And the same way on the $p_{g}^{n}$, which is the global optimal solution of all particles until iteration *n*.
**9.** **Stop condition checking:** If the maximum iterations predefined are met, go to step **3**. Otherwise, go to step **10**.
**10.** To avoid overtraining, stop training when the iteration has the best CV accuracy.
**11.** Build the SVM model on the verification samples based on the SVM model optimal parameters (*C*, *γ*), which are obtained with the stopping iteration determined in the step **10**.
**12.** End the training and verification procedure and get the result of the PSO-SVM model.

**Table 2:** Procedures of the GWR-PSO-SVM algorithm.

Input: Ancillary Data of the Study Area.
Output: The Landslide Susceptibility Map.
**Step 1: Extract environmental factors** ✓ Extract environmental factors from ancillary data, including digital elevation models, geological maps, topographical maps and remote sensing images, *etc.* Note that all data should be resampled to the same spatial resolution. ✓ To each computing unit, a value is assigned to represent its corresponding environmental factor.
**Step 2: Environmental factors screening** ✓ Calculate the Pearson product-moment correlation coefficient (PPMCC) between any pair of environmental factors and exclude the environmental factors with high correlations. If the PPMCC value is greater than a predefined threshold *T*_1_, the corresponding environmental factors are excluded according to the actual situation of the study area and previous research works. ✓ Calculate the importance value in the SVM model for each remaining environmental factor. In this work, the importance values, which are greater than a predefined threshold *T*_2_, are preserved as the final ones for the corresponding environmental factors. Finally, these environmental factors are used for the subsequent landslide prediction.
**Step 3: Study area segmentation** ✓ Select an appropriate kernel function and information criterion method according to Equations (4) and (5), respectively. ✓ Calculate a GWR coefficient for each computing unit of each environmental factor according to Equations (1)–(3) by inputting the geographic coordinates of each center point and the values of all computing unit mentioned in **Step 1**. ✓ Divide each environmental factor into *N* classes using the natural breaks method based on GWR coefficient values. In this work, *M* environmental factors, which are determined in **Step 1**, are chosen for study area segmentation. As a result, *M* classification maps are produced. ✓ Superpose all the classification maps to obtain a superposed map and merge very small regions in this map to generate a final prediction region map according to Figure 6.
**Step 4: The PSO-SVM prediction** ✓ To perform SVM prediction, training samples are constructed by using all the computing units with landslide and the same number of randomly selected computing units without landslide. ✓ The two-class SVM classifier with the Gaussian RBF kernel is used for prediction. Then, perform the PSO algorithm to obtain the optimal C and *γ* for the SVM prediction model for each prediction region. Meanwhile, all the computing units are used for landslide susceptibility mapping according to Equation (8). In the resultant map, the probability values ranging from 0 to 100% are employed for representing different degrees of landslide susceptibility. ✓ Merge the result of each prediction region. All of computing units in the prediction regions without landslide are assigned to zero. Eventually, the final landslide susceptibility map of the study area is produced.

**Table 3:** Classification of the bedding structure.

Type	Definition
Over-dip slope	$\left|\alpha -\beta \right|\in \left[0^{\circ },30^{\circ }\right)\;or\;\left|\alpha -\beta \right|\in \left[330^{\circ },360^{\circ }\right),\gamma >10^{\circ }\;and\;\delta >\gamma$
Under-dip slope	$\left|\alpha -\beta \right|\in \left[0^{\circ },30^{\circ }\right)\;or\;\left|\alpha -\beta \right|\in \left[330^{\circ },360^{\circ }\right),\gamma >10^{\circ }\;and\;\delta <\gamma$
Dip-oblique slope	$\left|\alpha -\beta \right|\in \left[30^{\circ },60^{\circ }\right)\;or\;\left|\alpha -\beta \right|\in \left[300^{\circ },330^{\circ }\right)$
Transverse slope	$\left|\alpha -\beta \right|\in \left[60^{\circ },120^{\circ }\right)\;or\;\left|\alpha -\beta \right|\in \left[240^{\circ },300^{\circ }\right)$
Anaclinal-oblique slope	$\left|\alpha -\beta \right|\in \left[120^{\circ },150^{\circ }\right)\;or\;\left|\alpha -\beta \right|\in \left[210^{\circ },240^{\circ }\right)$
Anaclinal slope	$\left|\alpha -\beta \right|\in \left[150^{\circ },210^{\circ }\right)$

*α*: Slope aspect; *β*: bed dip direction; *γ*: bed dip angle; *δ*: slope angle.

**Table 4:** Landslide environmental factors and their respective values.

Environmental Factors		Value
Geomorphology	Elevation (m)	124.2727–922.3077
	Slope angle (°)	3.2045–36.2898
	Slope aspect (°)	28.4827–321.5051
	Terrain surface convexity (°/100m)	0.5979–0.2449
	Plane curvature (°/100m)	−0.4023–0.4832
	Profile curvature (°/100m)	−1.2441–1.2856
	Slope form	(1) V/V; (2) GE/V; (3) X/V; (4) V/GR; (5) GE/GR; (6) X/GR; (7) V/X; (8) GE/X; (9) X/X
	Slope height (m)	374.6390–3.6325
	Mid-slope position	0.1272–0.9491
	Terrain surface texture	0.8495–0.3018
	Terrain roughness index	1.1589–16.4521
	Terrain convergence index	−27.6027–19.7669
	Terrain curvature (°/100m)	−1.5762–1.4682
	Terrain position index	−14.6285–9.5591
Geology	Lithology	(1) mudstone, shale and Quaternary deposits; (2) sandstones and thinly bedded limestones; (3) limestones and massive sandstones
	Bedding structure	(1) over-dip slope; (2) under-dip slope; (3) dip-oblique slope; (4) transverse slope; (5) anaclinal-oblique slope; (6) anaclinal slope
Hydrology	Catchment area (m^2^)	1156.0378–105,783.4666
	Catchment slope (°)	0.0485–0.5675
	Flow path length (m)	50.1196–2352.5587
	Valley depth (m)	3.4642–258.2873
	Stream power index	−617,299.4571–281,486.9383
	Distance from drainage (m)	18.4328–5637.6471
	Topographic wetness index	8.2193–14.7816
	Vertical distance to channel network (m)	−184.3475–461.4196
Land cover	Land-use	(1) water; (2) residential; (3) forest; (4) agriculture; (5) grassland
	NDVI	−0.4856–0.8337
	NDWI	0.0206–0.69411
Meteorology	Precipitation (mm)	1134.0551–1192.7400
Geophysics	Magnitude (Ms)	1.2617–2.1209

**Table 5:** Correlations of geomorphological factors.

Environmental Factor	ELE	SLAN	SLAS	SLHE	SLFO	TST	TRI	TPI	TCI	MSLP	PLCU	PRCU	TCU	TSC
**ELE**	1													
**SLAN**	0.198	1												
**SLAS**	0.022	−0.099	1											
**SLHE**	0.321	0.581	0.03	1										
**SLFO**	−0.013	0.093	0.16	0.206	1									
**TST**	−0.255	−0.739	0.045	−0.562	−0.022	1								
**TRI**	0.188	0.995	−0.105	0.579	0.091	−0.735	1							
**TPI**	0.125	0.138	0.122	0.338	0.761	−0.062	0.133	1						
**TCI**	0.117	0.054	0.221	0.241	0.787	−0.013	0.047	0.810	1					
**MSLP**	0.08	0.007	0.015	0.176	−0.163	−0.143	0.025	−0.15	−0.16	1				
**PLCU**	−0.103	0.112	0.187	0.162	0.735	−0.052	0.114	0.601	0.641	−0.093	1			
**PRCU**	−0.172	−0.103	−0.08	−0.224	−0.564	0.017	−0.095	−0.809	−0.661	0.14	−0.3	1		
**TCU**	0.071	0.131	0.155	0.243	0.782	−0.04	0.127	0.889	0.804	−0.15	0.728	−0.872	1	
**TSC**	0.083	0.155	−0.015	0.356	0.169	0.172	0.142	0.204	0.165	0.021	0.034	−0.2	0.161	1

ELE = elevation, SLAN = slope angle, SLAS = slope aspect, SLHE = slope height, SLFO = slope form, TST = terrain surface texture, TRI = terrain roughness index, TPI = terrain position index, TCI = terrain convergence index, MSLP = mid-slope position, PLCU = plane curvature, PRCU = profile curvature, TCU = terrain curvature, TSC = terrain surface convexity.

**Table 6:** Correlations of hydrological factors.

Environmental Factor	DISD	CMA	FPL	TWI	VADE	CMSL	SPI	VDCN
**DISD**	1							
**CMA**	0.011	1						
**FPL**	−0.109	0.551	1					
**TWI**	−0.026	0.607	0.545	1				
**VADE**	−0.112	0.678	0.675	0.65	1			
**CMSL**	−0.007	0.327	0.41	0.411	0.638	1		
**SPI**	−0.055	−0.013	0.004	−0.112	−0.052	−0.004	1	
**VDCN**	−0.368	0.259	0.424	0.222	0.475	0.292	0.045	1

DISD = distance from drainage, CMA = catchment area, FPL = flow path length, TWI = topographic wetness index, VADE = valley depth, CMSL = catchment slope, SPI = stream power index, VDCN = vertical distance to channel network.

**Table 7:** The numbers of the total slope-units and the landslide slope-units for each prediction region.

Region ID	Number of Slope-Units	Number of Landslide Slope-Units	Region ID	Number of Slope-Units	Number of Landslide Slope-Units
1	59	9	18	75	18
2	51	5	19	40	0
3	8	2	20	63	14
4	59	0	21	52	12
5	52	5	22	54	12
6	17	0	23	52	13
7	61	19	24	57	15
8	61	0	25	71	24
9	138	29	26	134	60
10	57	9	27	10	0
11	38	12	28	80	36
12	80	0	29	9	0
13	21	2	30	76	12
14	90	23	31	7	0
15	64	8	32	47	22
16	77	14	33	70	31
17	42	0	34	37	10

**Table 8:** The parameter settings of *C* and *γ* calculated by the PSO algorithm for the GWR-PSO-SVM model.

**GWR-PSO-SVM Model**	**Region ID**	***C***	***γ***	**Region ID**	***C***	***γ***
	1	6.1826	0.13879	20	5.9453	0.29134
	2	1.2965	0.32455	21	5.3659	0.38439
	3	2.4682	0.31596	22	3.3548	0.17105
	5	1.4832	0.36957	23	5.8234	0.36851
	7	8.6235	0.51243	24	2.1629	0.47592
	9	4.1356	0.67572	25	3.2592	0.45665
	10	2.3659	0.49986	26	6.5359	0.67853
	11	2.6971	0.33645	28	6.2157	0.47935
	13	4.3651	0.42631	30	7.2853	0.63428
	14	5.8652	0.42375	32	6.4075	3.35874
	15	1.4964	0.56916	33	5.3364	0.47516
	16	4.7569	0.32793	34	4.8435	0.67203
	18	1.4259	0.47157	-		

**Table 9:** The training and verification sample of the five models.

Model	Region ID	Training Sample	Verification Sample	Region ID	Training Sample	Verification Sample
GWR-PSO-SVM and GWR-SVM	1	18	59	20	28	63
	2	10	51	21	24	52
	3	4	8	22	24	54
	5	10	52	23	26	52
	7	38	61	24	30	57
	9	58	138	25	48	71
	10	18	57	26	120	134
	11	24	38	28	72	80
	13	4	21	30	24	76
	14	46	90	32	44	47
	15	16	64	33	62	70
	16	28	77	34	20	37
	18	36	75			
SVM		832	1909			
PSO-SVM		832	1909			
RS-SVM		832	1909			

**Table 10:** Overall accuracies by all the prediction models.

Model	Correct	Total	Accuracy
SVM	1415	1909	74.12%
PSO-SVM	1590	1909	83.29%
RS-SVM	1427	1909	74.75%
GWR-SVM	1140	1584	71.97%
GWR-PSO-SVM	1443	1584	*91.10%*

**Table 11:** The AUC of four models.

Model	Area	Std. Error	Asymptotic Sig.	Asymptotic 95% Confidence Interval	
				Lower Bound	Upper Bound
SVM	0.817	0.011	0.000	0.796	0.837
PSO-SVM	0.869	0.010	0.000	0.850	0.889
RS-SVM	0.825	0.010	0.000	0.804	0.845
GWR-SVM	0.860	0.009	0.000	0.842	0.878
GWR-PSO-SVM	0.971	0.004	0.000	0.963	0.978

Std. = Standard; Sig. = Significant.
